# Synthesis and Biological Evaluation of Novel Benzimidazole Derivatives and Analogs Targeting the NLRP3 Inflammasome

**DOI:** 10.3390/molecules22020213

**Published:** 2017-01-30

**Authors:** Liangkun Pan, Nan Hang, Chao Zhang, Yu Chen, Shuchun Li, Yang Sun, Zhongjun Li, Xiangbao Meng

**Affiliations:** 1 State Key Laboratory of Natural and Biomimetic Drugs, Department of Chemical Biology, School of Pharmaceutical Sciences, Peking University, Beijing 100191, China; panliangkun1012@163.com (L.P.); CHZ62@pitt.edu (C.Z.); jmc610@126.com (Y.C.); scli@bjmu.edu.cn (S.L.); zjli@bjmu.edu.cn (Z.L.); 2 State Key Laboratory of Pharmaceutical Biotechnology, School of Life Sciences, Nanjing University, Nanjing 210093, China; hangnan_1991@163.com

**Keywords:** benzimidazole, NLRP3, IL-1β, anti-inflammatory, drug discovery

## Abstract

A series of benzo[*d*]imidazole analogues of thiabenzole were synthesized and their anti-inflammatory activities toward NLRP3 (nucleotide-binding domain leucine-rich repeat containing protein family, pyrin domain-containing 3, also known as cryopyrin or NALP3) inflammasome were evaluated in vitro. Two lead compounds, **TBZ-09** and **TBZ-21**, were identified by anti-production of IL-1β. In the second round of biological evaluation, based on the lead, 34 more compounds were synthesized and their in vitro anti-inflammatory activities were investigated. Several compounds were identified as anti-inflammatory agents that can reduce IL-1β expression in a dose-dependent manner. A preliminary structure–activity relationship is also summarized here.

## 1. Introduction

The natural immune system typically senses infection or injury through pattern recognition receptors, collectively called pathogen-associated molecular patterns (PAMPs) shared by groups of different microbial pathogens and recognized by toll-like receptors (TLRs) or other receptors expressed on the cell surface of immune cells, and responds by secreting cytokines and chemokines. One of the most important inflammatory cytokines secreted by macrophages is IL-1β, a potent pro-inflammatory cytokine that contributes to host defense and the pathogenesis of several inflammatory diseases [1]. Consequently, IL-1β secretion is tightly regulated at both the translational and post-translational levels. Unlike other cytokines, IL-1 is synthesized by phagocytes as an inactive and immature precursor which is processed into biologically active form IL-1β by the inflammasome, a multi-protein complex [2]. Inflammasome activation results in the recruitment and activation of caspase-1, which then cleaves pro-IL-1β and pro-IL-18 into their mature forms. Inflammasomes consist of a sensor protein, which can be a Nod2-like receptor, such as NLRP3, NLRP1, and NLRC4, or the PYHIN family member AIM2 (absent in melanoma 2) [3]. The NLRP3 inflammasome is particularly interesting among the inflammasomes because it controls disease progression and inflammatory responses, such as those caused by bacteria, viruses, components released by dying cells, and particulate matter [4]. NLRP3 has been implicated in the pathogenesis of several human diseases, such as gouty arthritis, silicosis, type 2 diabetes, atherosclerosis, and Alzheimer disease [5,6,7,8,9,10,11,12]. Although the NLRP3 inflammasome has been intensively investigated using cell culture, mouse genetic models, and various infection models, the signaling mechanism leading to NLRP3 inflammasome activation is still unclear [12]. In addition, there are still limited medicinal chemistry or chemical biology researches on NLRP3 inflammasome-based drug discovery. Therefore, to gain a better insight to the signaling mechanism of NLRP3 inflammasome activation will enable the development of novel therapeutic strategies to treat NLRP3-associated human diseases. Thus, it is of great meaning to develop novel anti-inflammatory drugs based on the NLRP3 inflammasome other than classical non-steroidal anti-inflammatory drugs (NSAIDs) and glucocorticoids that may cause so many side effects like gastrointestinal irritation and withdrawal syndrome. However, it is still the challenge of the pharmaceutical chemist to develop more effective and less toxic agents to treat the signs and symptoms of acute inflammation as well as the long-term consequences of chronic inflammatory diseases [13].

**Figure 1:**
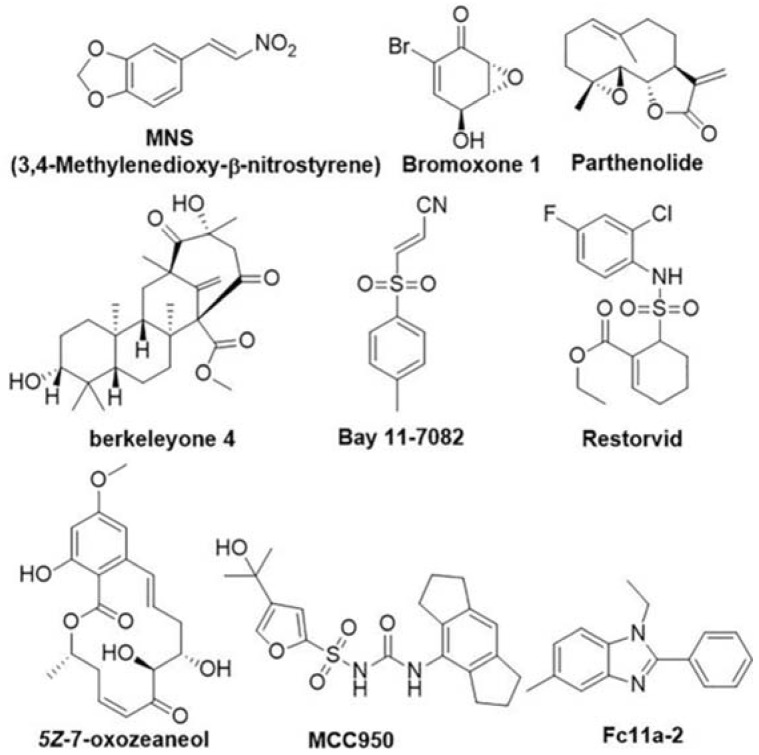
Recently published NLRP3 inflammasome inhibitors.

As shown in Figure 1, small molecules such as MNS [14], Bromoxone 1 [15], parthenolide [16], Bay 11-7082 [16], Restorvid [15], 5z-7-oxozeaneol [15], and compound **9** [17], are potent NLRP3 inflammasome inhibitors reported at least partly due to their special Michael addition acceptor activities. Additionally, other small molecules are identified to be negative regulatorsof NLRP3 inflammasome activation, among them berkeleyone 4 [18] and MCC-950 [19] are excellent examples. MCC-950 has also been verified to be the most potent inhibitor targeting the NLRP3 inflammasome, with IC_50_ of ~3 nM against IL-1β secretion in vitro. These NLRP3 inflammasome-targeting agents provided us with a molecular rationale for future therapeutic intervention in inflammation-related diseases. Our previous investigation verified compound 1-ethyl-5-methyl-2-phenyl-1*H*-benzo[*d*] imidazole (Fc11a-2) as a novel NLRP3 inflammasome inhibitor for the treatment of murine experimental colitis caused by dextran sulfate sodium (DSS) [20]. Recently we have synthesized a series of thiabendazole analogs as novel anti-angiogenic and vascular disrupting agents [21], it was logical to deduce that they would also act on the NLRP3 inflammasome based on their structure similarity. Herein, two series of compounds bearing benzimidazole core or its scaffold mimics were synthesized, screened by the IL-1β production level on LPS activated macrophages. Several compounds were identified as anti-inflammatory agents that can reduce IL-1β expression in a dose-dependent manner. A preliminary structure–activity relationship was also summarized.

## 2. Results and Discussion

### 2.1. Synthesis

**Scheme 1:**
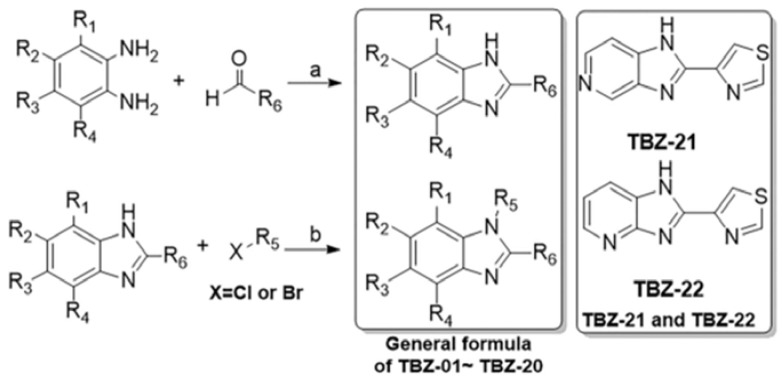
Synthesis of compounds **TBZ-01**–**TBZ-22** *Reagents and conditions*: (**a**) Na_2_S_2_O_5_, DMF, 120 °C; 51%–85%; and (**b**) NaH, DMF, r.t. 68%–92%.

The first round synthesis of 22 compounds (Scheme 1 and Table 1) based on thiabenzole core was accomplished by condensations of *o*-phenyldiamine derivatives with thiazole-4-aldehyde and pydrine-2-aldehyde catalyzed by sodium pyrosulfite in DMF under 120 °C, respectively [21,22]. Two lead compounds, **TBZ-09** and **TBZ-21**, were identified after the biological evaluation of IL-1β secretion (Figure 3). Clearly, both compounds share a common characteristic that they have strong electronic-withdrawing group on the benzene ring.

**Figure 2:**
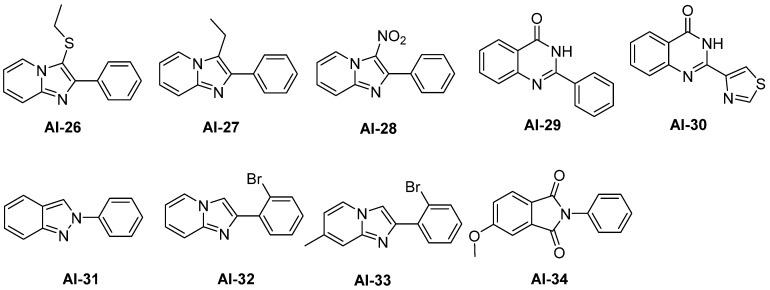
Benzimidazole scaffold hopping compounds.

Taking advantages of the preliminary SAR we obtained from the first round biological activity screening, we move on to synthesize another series of compounds, which is shown in Table 2. We synthesized **AI-22** and **AI-23** with benzyl substitution at the N-1 position to test their activity. In addition, by means of scaffold hopping, we devised and synthesized several compounds, which are listed in Figure 2 as benzimidazole mimics. We either replaced carbon atom by nitrogen in the benzimidazole core, **AI-26**, **AI-27**, **AI-28**, **AI-31**, **AI-32**, and **AI-33**, or enlarge the ring of former imidazole part, **AI-29** and **AI-30** [23,24,25,26,27,28]. The side chain of imidazole was also replaced by sulfur atom, etc. Due to their structure diversity, we described their methods of synthesis in the Experiment Section in detail.

### 2.2. Biology

#### In Vitro IL-1β Production Regulated by Compounds

To seek for small molecules with anti-inflammatory activities in vitro, we screened a series of synthetic compounds by assaying their inhibitory activities of IL-1β, which was produced by THP-1 cells activated by lipopolysaccharide (LPS, priming signal) and ATP. Many microbial Toll-like receptor ligands, such as lipopolysaccharide, have been shown to prime cells by including the transcription and translation of NLRP3 protein itself [6,29,30]. A further signal (activating signal), such as ATP or nigericin, is required to trigger the formation of the inflammasome complex that leads to the activation of caspase-1 and release of cytokines such as IL-1β, IL-18, etc.

**Figure 3:**
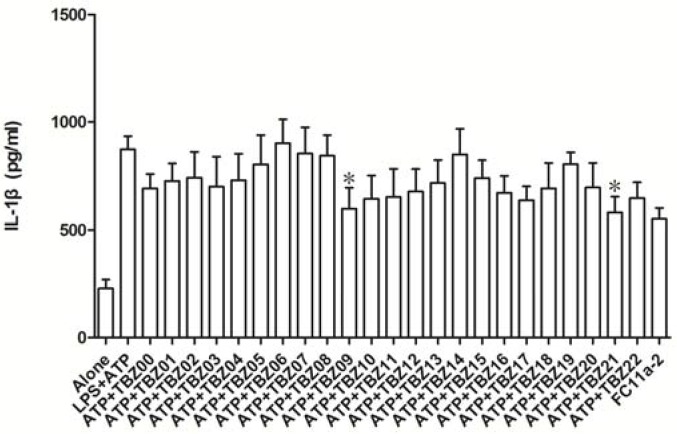
The effects of TBZ series compounds (10 μM) on IL-1β productions in THP-1 cells activated by LPS plus ATP. (The marked compounds exhibit the best inhibition towards IL-1β production.)

As shown in Figure 3, compared to the control group (LPS + ATP), most compounds exhibited inhibitory effect of IL-1β release in varying degrees except for **TBZ-06**. Among all these compounds, **TBZ-09** and **TBZ-21** showed the most potent activities against IL-1β release in vitro. Therefore, a preliminary SAR was summarized based on above data. **TBZ-09** and **TBZ-21** actually shared the common characteristic that they both bear an electron-withdrawing group at 5 position of the benzimidazole core (for **TBZ-09**, a nitro group, and for **TBZ-21**, a nitrogen atom). In addition, benzyl substitution at the 1 position, such as **TBZ-11**, helps enhance the potency. The abovementioned SAR thus provided us a theoretical basis for further derivatization.

**Figure 4:**
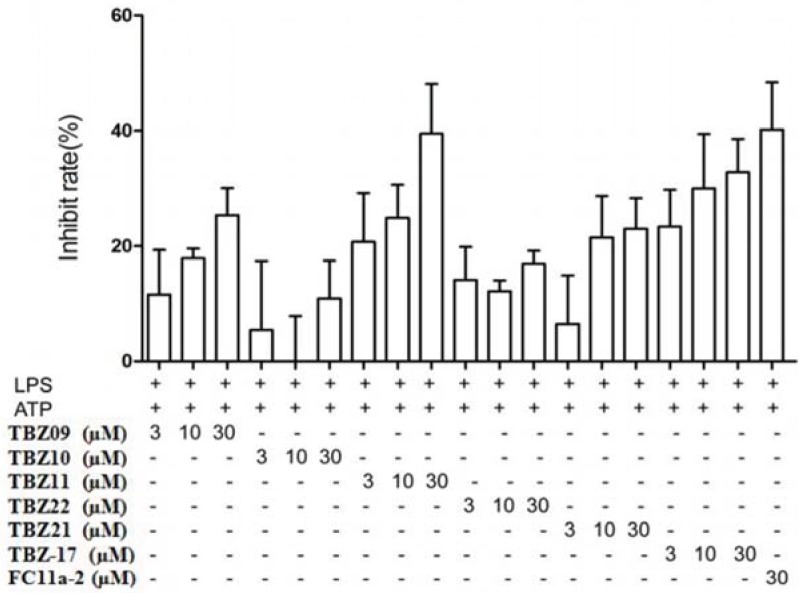
The effects of various doses of **TBZ-09**, -**10**, -**11**, -**17**, -**21**, and -**22** on IL-1β productions in THP-1 cells activated by LPS plus ATP.

We then select compounds **TBZ-09**, -**10**, -**11**, -**17**, -**21**, and -**22** for further investigation. As shown in Figure 4, **TBZ-09** and **TBZ-21** exhibit moderate inhibition towards IL-1β production in a dose-dependent manner. The maximum inhibitory rate could be about 30% at 30 μM. In addition, **TBZ-11** could remarkably inhibit the release of IL-1β in a dose-dependent manner. The inhibitory rate could be as high as 40%, which is similar to Fc11a-2 in potency. We then synthesized compounds of AI series (Table 2) bearing electron-withdrawing group at 5 position in the same pattern with **TBZ-09** and **TBZ-21**. Some compounds were prepared with a benzyl substitution at the N position of benzimzazole (**AI-22** and **AI-23**).

**Figure 5:**
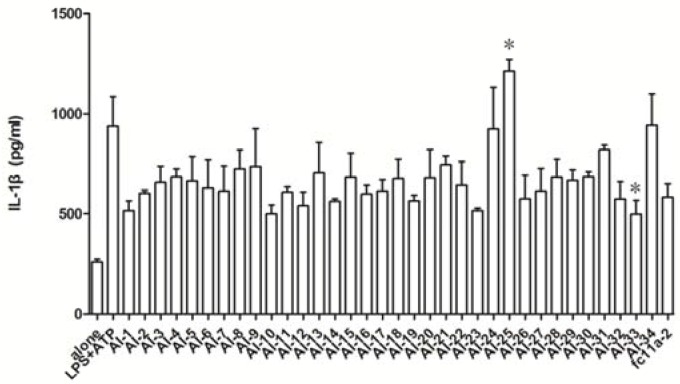
The effects of AI series compounds (10 μM) on IL-1β productions in THP-1 cells activated by LPS plus ATP. (The marked compound AI-25 exhibits the best activating activity towards IL-1β production, while compound AI-33 exhibits the best inhibition activity.)

The anti-inflammatory effects of AI compounds on IL-1β release were evaluated in vitro. As shown in Figure 5, compared with Fc11a-2, compounds like **AI-1**, -**10**, -**12**, -**14**, -**19**, -**23**, -**26**, -**32**, and -**33** exhibited slightly improved potency. To our surprise, compounds **AI-24**, -**25**, and -**34** showed significantly potent IL-1β activating activity. Among them, **AI-25** could almost double the release amount of IL-1β, which seemed to act in a different mechanism from others. In addition, to our knowledge, there are no reports about medical applications about improving cytokines like IL-1β. Taken together, we identified that different substituted groups in the 2-aryl parts showed limited improvement in potency, whether they are electron-withdrawing groups like nitro group, fluoride group, electron-donating groups like the methoxyl group, *N*,*N*-dimethyl group or other heterocycles like furan.

## 3. Experiment Sections

### 3.1. Chemistry

#### 3.1.1. General

Unless otherwise noted, all reagents were obtained from commercial suppliers and used without further purification. Reactions were monitored by thin-layer chromatography (TLC) using commercial silica gel HSGF254 plates. Column chromatography was performed on Silica Gel 60 (E. Merck, 230–400 mesh, Darmstadt, Germany). Melting points were measured on an X-5 micromelting point apparatus (Yuezhong Instruments Co., Ltd., Shanghai, China) and were uncorrected. The ^1^H-MMR (400 MHz) and ^13^C-NMR (100.6 MHz) spectra were recorded with BrukerAM-400 spectrometer (Bruker Co., Ltd., Zurich, Switzerland) in CDCl_3_ and DMSO-*d*_6_ solution. Chemical shifts were referenced with tetramethylsilane (TMS). The HR-ESI-MS data were measured on a Bruker Apex IV FTMS (Bruker Co., Ltd., Karlsruhe, Germany).

**TBZ-1**–**20** were synthesized recently [21], and **AI-1**–**18**, and **AI-22**–**25** were prepared using a similar method. Spectral characterization details of all compounds are available in the Supplementary Materials.

#### 3.1.2. Procedure for the Synthesis of **AI-19**

To a solution of 4-amino-3-nitropyridine (2.0 mmol) in DMF (10 mL), NaH (2.2 mmol) was added slowly, the mixture was stirred at room temperature for 30 min. Then, allyl bromide (2.0 mmol) was added slowly into the mixture until the reaction is finished monitored by TLC. Then the solvent was evaporated and the residue was purified by silica gel chromatography by DCM/MeOH system to afford 4-allylamino-3-nitropydine.

To a solution of 4-allylamino-3-nitropydine (1.0 mmol) in the mixture of HOAc/H_2_O (5 mL:5 mL), Fe powder (5.0 mmol) was added, then the mixture was refluxed at 100 °C until the completion of the reaction. Then the solvent was evaporated, residue washed by saturated NaHCO_3_ solution, extracted by DCM for three times. The organic layer was combined, the residue was purified by DCM/MeOH system to afford 4-allylamino-3-aminopydine.

The solution of substituted 4-allylamino-3-aminopydine (0.5 mmol), thiazole-4-aldehyde (0.5 mmol) with sodium pyrosulfite (0.5 mmol) in DMF (8 mL) was stirred under 120 °C overnight. On completion of the reaction, the solvent was evaporated and the residue was purified by silica gel chromatography by DCM/MeOH system to afford the final product. If necessary, the crude product could be recrystallized in DCM to afford pure compound.

#### 3.1.3. Procedure for the Synthesis of **AI-20**

To a solution of 4-amino-3-nitropyridine (2.0 mmol) in DMF (10 mL), NaH (2.2 mmol) was added slowly, the mixture was stirred at room temperature for 30 min. Then ethyl iodine (2.0 mmol) was added slowly into the mixture until the reaction is finished monitored by TLC. Then the solvent was evaporated and the residue was purified by silica gel chromatography by DCM/MeOH system to afford 4-ethylamino-3-nitropydine.

To a solution of 4-ethylamino-3-nitropydine (1.0 mmol) in EtOH (10 mL), 10% Pd/C was added. Then the mixture was reduced by catalytic hydrogenation. Until the completion of the reaction, the Pd/C was filtered and the solvent was evaporated. The product was used in the next step without further purification.

The solution of substituted 4-ethylamino-3-aminopydine (0.5 mmol), benzaldehyde (0.5 mmol) with sodium pyrosulfite (0.5 mmol) was stirred in DMF (8 mL) under 120 °C overnight. On completion of the reaction, the solvent was evaporated and the residue was purified by silica gel chromatography by DCM/MeOH system to afford the final product. If necessary, the crude product could be recrystallized in DCM to afford pure compound [31].

#### 3.1.4. Procedure for the Synthesis of **AI-21**

To a solution of 4-amino-3-nitropyridine (2.0 mmol) in DMF (10 mL), NaH (2.2 mmol) was added slowly, the mixture was stirred at room temperature for 30 min. Then ethyl iodine (2.0 mmol) was added slowly into the mixture until the reaction is finished monitored by TLC. Then the solvent was evaporated and the residue was purified by silica gel chromatography by DCM/MeOH system to afford 4-ethylamino-3-nitropydine.

To a solution of 4-ethylamino-3-nitropydine (1.0 mmol) in EtOH (10 mL), 10% Pd/C was added. Then the mixture was reduced by catalytic hydrogenation. Until the completion of the reaction, the Pd/C was filtered and the solvent was evaporated. The product was used in the next step without further purification.

The solution of substituted 4-ethylamino-3-aminopydine (0.5 mmol), thiazaole-4-aldehyde (0.5 mmol) with sodium pyrosulfite (0.5 mmol) in DMF (8 mL) was stirred under 120 °C overnight. On completion of the reaction, the solvent was evaporated and the residue was purified by silica gel chromatography by DCM/MeOH system to afford the final product. If necessary, the crude product could be recrystallized in DCM to afford pure compound.

#### 3.1.5. Procedure for the Synthesis of **AI-26**

The acetophenone (0.5 mmol), 2-aminopyridine (0.5 mmol), and ethyl sulfide (0.25 mmol) were dissolved in EtOH (1 mL) at 80°C in a 35 mL sealed tube, and then CeCl_3_·7H_2_O/NaI (0.05 mmol, 10 mol %) was added. The reaction proceeded under an O_2_ atmosphere for the indicated time until complete consumption of the starting material as indicated by TLC. The solution was diluted with EtOAc (10 mL) and washed with H_2_O (3 × 10 mL). Then, the organic layer was separated and concentrated under vacuum, and the crude product was purified by column chromatography (petroleum ether/EtOAc, 5:1) to provide pure **AI-26** [23].

#### 3.1.6. Procedure for the Synthesis of **AI-27**

The butyrophenone (0.5 mmol) and 2-aminopyridine (0.5 mmol) were dissolved in EtOH (1 mL) at 80 °C in a 35 mL sealed tube, and then CeCl_3_·7H_2_O/NaI (0.05 mmol, 10 mol %) was added. The reaction proceeded under an O_2_ atmosphere for the indicated time until complete consumption of the starting material as indicated by TLC. The solution was diluted with EtOAc (10 mL) and washed with H_2_O (3 × 10 mL). Then, the organic layer was separated and concentrated under vacuum, and the crude product was purified by column chromatography (petroleum ether/EtOAc) to provide the final product **AI-27** [24].

#### 3.1.7. Procedure for the Synzthesis of **AI-28**

The solution of trans-nitrostyrene (0.6 mmol), 2-aminopyridine (0.5 mmol) and TBAI (0.1 mmol) in DMF 2 mL was added TBHP (1.0 mmol, 70% aq solution). The reaction was stirred at 80 °C for 4 h. Once the reaction was completed, it was diluted with DCM, washed with brine, and dried over MgSO_4_. After the solvent was evaporated in vacuo, the residues were purified by column chromatography, eluted with PE:EtOAc to afford the final product **AI-28** [25].

#### 3.1.8. General Procedure for the Synthesis of **AI-29** and **AI-30**

To a stirred mixture of anthranilamide (20 mmol) and benzaldehyde (20 mmol) or thiazole-4-carboxaldehyde (20 mmol), respectively, in 20 mL DMF, iodine (3.17 g, 25 mmol) and anhydrous potassium carbonate (2.76 g, 20 mmol) were added. Then the mixture was heated at 70–90 °C for 4–5 h. The mixture was poured on to crushed ice and the precipitate formed was filtered. The product was washed with 100 mL 20% solution of sodium thiosulfate to remove traces of iodine, followed by washing with water. The product was recrystallized from ethanol to yield pale yellow to dark yellow crystals [26].

#### 3.1.9. Procedure for the Synthesis of **AI-31**

The Cu_2_O catalyst (0.1 mmol) was added to a solution of 2-bromobenzaldehyde (1.0 mmol), amine (1.0 mmol) and NaN_3_ (2.0 mmol) in polyethylene glycol (PEG300, 3.0 mL). The mixture was stirred at 120 °C. After completion of the reaction, the reaction mixture was cooled to r.t., poured into EtOAc and was washed with water and brine. The organic layer was dried, filtered and evaporated in vacuo to give the crude product, which was further purified by silica gel chromatography to afford the final product [28].

#### 3.1.10. General Procedure for the Synthesis of **AI-32** and **AI-33**

A Schlenk tube equipped with a stirrer bar was charged with 2-bromoacetophenone (2 mmol), 4-methyl-2-aminopyridine or 2-aminopyridine (6 mmol), CuI (0.4 mmol), and BF_3_·Et_2_O (0.2 mmol). The Schlenk tube was quickly evacuated, closed under vacuum, and then refilled with oxygen using an oxygen balloon. The resulting mixture was stirred at 40 °C for 24 h. After the completion of reaction, the residue was directly purified by flash column chromatography using ethyl acetate and petroleum ether as eluents to afford the pure product [32].

#### 3.1.11. Procedure for the Synthesis of **AI-34**

The solution of 4-methoxyphthalic acid (16 mmol) and Ac_2_O (0.4 mL) in 2 mL THF was refluxed for 4 h. Once completed, the solvent was evaporated and the intermediate 4-methyl phthalic anhydride was afforded. Then it was mixed with aniline (0.16 mL) in 5 mL acetic acid under reflux to afford the final product quantitively [27].

*2-Phenyl-1H-imidazo[4,5-c]pyridine* (**AI-1**). White solid; yield 81%; ^1^H-NMR (400 MHz, DMSO-*d*_6_) δ 13.36 (s, 1H), 8.95 (s, 1H), 8.32 (d, *J* = 5.5 Hz, 1H), 8.23 (d, *J* = 6.8 Hz, 2H), 7.64–7.52 (m, 4H).

*2-(4-Fluorophenyl)-1H-imidazo[4,5-c]pyridine* (**AI-2**). White solid; yield 72%; m.p. >200 °C; ^1^H-NMR (400 MHz, DMSO-*d*_6_) δ 13.34 (s, 1H), 8.94 (s, 1H), 8.34–8.22 (m, 3H), 7.59 (d, *J* = 5.3 Hz, 1H), 7.43 (t, *J* = 8.8 Hz, 2H); ^13^C-NMR (101 MHz, DMSO-*d*_6_) δ 165.21, 162.74, 153.42, 141.60, 129.87, 129.79, 126.66, 116.69, 116.48; HR-EI-MS: Calcd. for C_12_H_8_FN_3_ [M + H]^+^: 214.07358; found: 214.07707.

*2-(Benzo[d][1,3]dioxol-5-yl)-1H-imidazo[4,5-c]pyridine* (**AI-3**). Off-white solid; yield 77%; m.p. >200 °C; ^1^H-NMR (400 MHz, DMSO-*d*_6_) δ 8.91 (s, 1H), 8.29 (d, *J* = 3.6 Hz, 1H), 7.86–7.65 (m, 2H), 7.56 (d, *J* = 4.2 Hz, 1H), 7.12 (d, *J* = 7.8 Hz, 1H), 6.14 (s, 2H); ^13^C-NMR (101 MHz, DMSO-*d*_6_) δ 149.88, 148.42, 141.41, 123.89, 122.19, 109.28, 107.32, 102.23; HR-EI-MS: Calcd. for C_13_H_9_N_3_O_2_ [M + H]^+^: 240.07283; found: 240.076315.

*2-(3, 4-Dimethoxyphenyl)-1H-imidazo[4,5-c]pyridine* (**AI-4**). Off-white solid; yield 72%; m.p. 199–202 °C; ^1^H-NMR (400 MHz, DMSO-*d*_6_) δ 8.91 (s, 1H), 8.29 (d, *J* = 5.4 Hz, 1H), 7.80 (d, *J* = 6.6 Hz, 2H), 7.57 (d, *J* = 5.4 Hz, 1H), 7.15 (d, *J* = 8.9 Hz, 1H), 3.87 (d, *J* = 16.3 Hz, 6H); ^13^C-NMR (101 MHz, DMSO-*d*_6_) δ 150.94, 148.96, 141.06, 121.84, 120.05, 111.84, 110.05, 55.66, 55.62; HR-EI-MS: Calcd. for C_14_H_14_N_3_O_2_ [M + H]^+^: 256.10413; found: 256.10757.

*2-(3,5-Difluorophenyl)-1H-imidazo[4,5-c]pyridine* (**AI-5**). Off-white solid; yield 83%; m.p. 204–206 °C; ^1^H-NMR (400 MHz, DMSO-*d*_6_) δ 13.52 (s, 1H), 8.99 (s, 1H), 8.32 (d, *J* = 5.5 Hz, 1H), 7.89 (d, *J* = 6.7 Hz, 2H), 7.64 (d, *J* = 5.4 Hz, 1H), 7.45 (t, *J* = 9.1 Hz, 1H); ^13^C-NMR (101 MHz, DMSO-*d*_6_) δ 164.00, 163.87, 161.55, 161.42, 134.82, 133.22, 125.41, 110.22, 109.94, 105.80; HR-EI-MS: Calcd. for C_12_H_8_F_2_N_3_ [M + H]^+^: 232.06808; found: 232.06801.

*4-(1H-Imidazo[4,5-c]pyridin-2-yl)-N,N-dimethylaniline* (**AI-6**). Pale yellow solid; yield 80%; m.p. >200 °C; ^1^H-NMR (400 MHz, DMSO) δ 8.83 (s, 1H), 8.24 (d, *J* = 5.0 Hz, 1H), 8.04 (d, *J* = 8.9 Hz, 2H), 7.50 (d, *J* = 5.3 Hz, 1H), 6.84 (d, *J* = 9.0 Hz, 2H), 3.02 (d, *J* = 10.5 Hz, 6H); ^13^C-NMR (101 MHz, DMSO) δ 151.69, 140.70, 128.12, 116.17, 111.73, 54.89, 45.54.

*2-(Furan-2-yl)-1H-imidazo[4,5-c]pyridine* (**AI-7**). Dark pale solid; yield 71%; m.p. >200 °C; ^1^H-NMR (400 MHz, DMSO-*d*_6_) δ 8.91 (s, 1H), 8.29 (t, *J* = 10.6 Hz, 1H), 8.00 (d, *J* = 0.9 Hz, 1H), 7.58 (t, *J* = 10.9 Hz, 1H), 7.32 (d, *J* = 3.3 Hz, 1H), 6.76 (dd, *J* = 3.4, 1.7 Hz, 1H); ^13^C-NMR (101 MHz, DMSO-*d*_6_) δ 146.13, 145.48, 145.02, 140.95, 138.57, 112.53, 112.21, 109.14; HR-EI-MS: Calcd. for C_10_H_8_N_3_O [M + H]^+^: 186.06267; found: 186.06583.

*2-(1H-Indol-3-yl)-1H-imidazo[4,5-c]pyridine* (**AI-8**). Off-white solid; yield 79%; m.p. >300 °C; ^1^H-NMR (400 MHz, DMSO-*d*_6_) δ 11.79 (s, 1H), 8.87 (s, 1H), 8.59–8.46 (m, 1H), 8.25 (s, 2H), 7.52 (dd, *J* = 5.9, 2.4 Hz, 2H), 7.23 (p, *J* = 7.7 Hz, 2H); ^13^C-NMR (101 MHz, DMSO-*d*_6_) δ 140.85, 136.57, 127.33, 125.19, 122.43, 121.30, 120.59, 112.07, 105.87; HR-EI-MS: Calcd. for C_14_H_11_N_4_ [M + H]^+^: 235; found: 235.09736.

*2-(1H-Indol-2-yl)-1H-imidazo[4,5-c]pyridine* (**AI-9**). Off-white solid; yield 71%; m.p. 185–187 °C; ^1^H-NMR (400 MHz, DMSO-*d*_6_) δ 12.14 (s, 1H), 9.00 (s, 1H), 8.32 (t, *J* = 23.5 Hz, 1H), 7.64 (dd, *J* = 22.2, 6.3 Hz, 2H), 7.52 (d, *J* = 8.0 Hz, 1H), 7.36 (s, 1H), 7.21 (t, *J* = 7.3 Hz, 1H), 7.06 (t, *J* = 7.2 Hz, 1H); ^13^C-NMR (101 MHz, DMSO-*d*_6_) δ 141.63, 137.98, 128.45, 128.24, 123.79, 121.56, 120.38, 112.61, 103.58; HR-EI-MS: Calcd. for C_14_H_11_N_4_ [M + H]^+^: 235.09390; found: 235.09742.

*6-Nitro-2-phenyl-1H-benzo[d]imidazole* (**AI-10**). Light yellow solid; yield 77%; ^1^H-NMR (400 MHz, DMSO-*d*_6_) δ 13.60 (s, 1H), 8.50 (s, 1H), 8.21 (d, *J* = 6.3 Hz, 2H), 8.13 (d, *J* = 8.3 Hz, 1H), 7.75 (s, 1H), 7.59 (d, *J* = 7.1 Hz, 3H).

*2-(4-Fluorophenyl)-6-nitro-1H-benzo[d]imidazole* (**AI-11**). Light yellow solid; yield 75%; ^1^H-NMR (400 MHz, DMSO-*d*_6_) δ 13.48 (s, 1H), 8.35 (s, 1H), 8.18 (dd, *J* = 8.5, 5.5 Hz, 2H), 8.03 (dd, *J* = 8.8, 1.9 Hz, 1H), 7.66 (d, *J* = 8.6 Hz, 1H), 7.37 (t, *J* = 8.8 Hz, 2H).

*2-(Benzo[d][1,3]dioxol-5-yl)-5-nitro-1H-benzo[d]imidazole* (**AI-12**). Orange solid; yield 71%; m.p. >200 °C; ^1^H-NMR (400 MHz, DMSO-*d*_6_) δ 8.35 (s, 1H), 8.05 (dd, *J* = 8.8, 2.2 Hz, 1H), 7.72 (dd, *J* = 8.2, 1.6 Hz, 1H), 7.65 (d, *J* = 8.6 Hz, 2H), 7.08 (d, *J* = 8.1 Hz, 1H), 6.13 (s, 2H); ^13^C-NMR (101 MHz, DMSO-*d*_6_) δ 156.06, 150.06, 148.41, 142.93, 123.42, 122.26, 118.19, 109.24, 107.20, 102.30; HR-EI-MS: Calcd. for C_14_H_9_N_3_O_4_ [M + H]^+^: 284.06266; found: 284.06629.

*2-(3,4-Dimethoxyphenyl)-5-nitro-1H-benzo[d]imidazole* (**AI-13**). Orange solid; yield 85%; ^1^H-NMR (400 MHz, CDCl_3_) δ 8.47 (s, 1H), 8.17 (d, *J* = 8.6 Hz, 1H), 7.75 (s, 1H), 7.65 (dd, *J* = 20.6, 8.5 Hz, 2H), 6.95 (d, *J* = 8.4 Hz, 1H), 3.92 (s, 3H), 3.85 (s, 3H).

*2-(3,5-Difluorophenyl)-5-nitro-1H-benzo[d]imidazole* (**AI-14**). Light yellow solid; yield 85%; ^1^H-NMR (400 MHz, DMSO-*d*_6_) δ 13.68 (s, 1H), 8.42 (s, 1H), 8.09 (d, *J* = 8.6 Hz, 1H), 7.82 (d, *J* = 6.2 Hz, 2H), 7.74 (d, *J* = 8.6 Hz, 1H), 7.45 (t, *J* = 8.4 Hz, 1H); ^13^C-NMR (101 MHz, DMSO-*d*_6_) δ 163.81, 161.49, 153.09, 142.91, 132.19, 118.22, 110.08, 109.80, 106.04; HR-EI-MS: Calcd. for C_13_H_8_F_2_N_3_O_2_ [M + H]^+^: 276.05791; found: 276.05787.

*N,N-Dimethyl-4-(5-nitro-1H-benzo[d]imidazol-2-yl)aniline* (**AI-15**). Orange solid; yield 75%; ^1^H-NMR (400 MHz, DMSO-*d*_6_) δ 13.19 (s, 1H), 8.33 (d, *J* = 65.7 Hz, 1H), 8.13–8.01 (m, 3H), 7.64 (d, *J* = 30.2 Hz, 1H), 6.84 (d, *J* = 8.9 Hz, 2H), 3.01 (s, 6H).

*2-(Furan-2-yl)-5-nitro-1H-benzo[d]imidazole* (**AI-16**). Off-white solid; yield 71%; m.p. >200 °C; ^1^H-NMR (400 MHz, DMSO-*d*_6_) δ 13.58 (s, 1H), 8.52–8.21 (m, 1H), 8.12–7.98 (m, 2H), 7.67 (s, 1H), 7.33 (d, *J* = 2.5 Hz, 1H), 6.77 (d, *J* = 1.5 Hz, 1H); ^13^C-NMR (101 MHz, DMSO-*d*_6_) δ 183.44, 182.09, 180.35, 155.65, 152.34, 150.30, 149.32; HR-EI-MS: Calcd. for C_11_H_7_N_3_O_3_ [M + H]^+^: 230.05210; found: 230.05564.

*2-(1H-Indol-3-yl)-5-nitro-1H-benzo[d]imidazole* (**AI-17**). Orange solid; yield 89%; ^1^H-NMR (400 MHz, DMSO-*d*_6_) δ 13.13 (s, 1H), 11.84 (s, 1H), 8.53–8.24 (m, 3H), 8.09 (d, *J* = 7.6 Hz, 1H), 7.66 (s, 1H), 7.53 (d, *J* = 8.5 Hz, 1H), 7.24 (dd, *J* = 9.2, 5.4 Hz, 2H).

*4-(1-Ethyl-1H-benzo[d]imidazol-2-yl)thiazole* (**AI-18**). Off-white solid; yield 71%; m.p. 140–142 °C; ^1^H-NMR (400 MHz, CDCl_3_) δ 8.92 (d, *J* = 1.9 Hz, 1H), 8.31 (d, *J* = 2.0 Hz, 1H), 7.80 (dd, *J* = 5.6, 3.2 Hz, 1H), 7.44 (dd, *J* = 5.9, 3.0 Hz, 1H), 7.36–7.27 (m, 2H), 4.79 (q, *J* = 7.1 Hz, 2H), 1.47 (t, *J* = 7.1 Hz, 3H); ^13^C-NMR (101 MHz, CDCl_3_) δ 152.96, 148.09, 146.53, 143.05, 135.55, 123.05, 122.65, 121.13, 119.79, 109.96, 40.23, 15.49.

*4-(1-Allyl-1H-imidazo[4,5-c]pyridin-2-yl)thiazole* (**AI-19**). Brown solid; yield 78%; m.p. 112–114 °C; ^1^H-NMR (400 MHz, DMSO-*d*_6_) δ 9.36 (d, *J* = 1.9 Hz, 1H), 9.03 (d, *J* = 17.8 Hz, 1H), 8.62 (d, *J* = 2.0 Hz, 1H), 8.38 (d, *J* = 5.6 Hz, 1H), 7.68 (t, *J* = 16.9 Hz, 1H), 6.02 (ddd, *J* = 22.3, 10.3, 5.2 Hz, 1H), 5.44 (d, *J* = 5.1 Hz, 2H), 5.09 (d, *J* = 10.3 Hz, 1H), 4.93 (dd, *J* = 17.2, 1.0 Hz, 1H); ^13^C-NMR (101 MHz, DMSO-*d*_6_) δ 155.68, 147.88, 145.98, 141.62, 141.57, 140.34, 139.64, 133.25, 123.91, 116.92, 106.49, 47.00.

*1-Ethyl-2-phenyl-1H-imidazo[4,5-c]pyridine* (**AI-20**). Orange solid; yield 66%; ^1^H-NMR (400 MHz, CDCl_3_) δ 9.15 (s, 1H), 8.48 (s, 1H), 7.73 (s, 2H), 7.56 (s, 3H), 7.39 (d, *J* = 4.0 Hz, 1H), 4.31 (d, *J* = 6.8 Hz, 2H), 1.47 (t, *J* = 6.4 Hz, 3H).

*4-(1-Ethyl-1H-imidazo[4,5-c]pyridin-2-yl)thiazole* (**AI-21**). Off-white solid; yield 86%; m.p. 171–172 °C; ^1^H-NMR (400 MHz, CDCl_3_) δ 9.08 (s, 1H), 8.93 (d, *J* = 1.7 Hz, 1H), 8.41 (t, *J* = 10.2 Hz, 1H), 8.35 (d, *J* = 1.8 Hz, 1H), 7.35 (d, *J* = 5.6 Hz, 1H), 4.77 (q, *J* = 7.1 Hz, 2H), 1.45 (t, *J* = 7.1 Hz, 3H); ^13^C-NMR (101 MHz, CDCl3) δ 153.36, 148.00, 147.26, 142.78, 142.15, 140.42, 140.27, 122.41, 105.32, 40.67, 15.46; HR-EI-MS: Calcd. for C_11_H_11_N_4_S [M + H]^+^: 231.06980; found: 231.06989.

*4-(1-Benzyl-5-nitro-1H-benzo[d]imidazol-2-yl)thiazole* (**AI-22**). Orange solid; yield 47%; m.p. >200 °C; ^1^H-NMR (400 MHz, DMSO-*d*_6_) δ 9.35 (d, *J* = 1.7 Hz, 1H), 8.70 (d, *J* = 1.7 Hz, 1H), 8.57 (d, *J* = 1.8 Hz, 1H), 8.15 (dd, *J* = 8.9, 1.9 Hz, 1H), 7.78 (d, *J* = 9.0 Hz, 1H), 7.24 (dq, *J* = 14.1, 7.0 Hz, 3H), 7.13 (d, *J* = 7.1 Hz, 2H), 6.20 (s, 2H); ^13^C-NMR (101 MHz, DMSO-*d*_6_) δ 156.03, 150.22, 145.70, 143.41, 141.73, 140.06, 136.74, 128.69, 127.57, 126.64, 124.91, 118.45, 115.20, 111.79, 48.11; HR-EI-MS: Calcd. for C_17_H_12_N_4_O_2_S [M + H]^+^: 337.07145; found: 337.07550.

*4-(1-Benzyl-6-nitro-1H-benzo[d]imidazol-2-yl)thiazole* (**AI-23**). Orange solid; yield 40%; m.p. 183–185 °C; ^1^H-NMR (400 MHz, DMSO-*d*_6_) δ 9.35 (d, *J* = 1.5 Hz, 1H), 8.72 (t, *J* = 8.2 Hz, 1H), 8.54 (d, *J* = 1.6 Hz, 1H), 8.13 (dd, *J* = 8.8, 1.8 Hz, 1H), 7.87 (d, *J* = 8.9 Hz, 1H), 7.31–7.21 (m, 3H), 7.14 (d, *J* = 7.2 Hz, 2H), 6.28 (s, 2H); ^13^C-NMR (101 MHz, DMSO-*d*_6_) δ 156.11, 151.20, 147.10, 145.76, 142.94, 136.86, 135.08, 128.70, 127.54, 126.57, 125.38, 119.39, 118.27, 108.10, 47.96; HR-EI-MS: Calcd. for C_17_H_12_N_4_O_2_S [M + H]^+^: 337.07145; found: 337.07526.

*2-(1H-Indol-3-yl)-1H-benzo[d]imidazole* (**AI-24**). Yellow solid; yield 73%; m.p. 233–235 °C; ^1^H-NMR (400 MHz, DMSO-*d*_6_) δ 12.98 (s, 1H), 12.01 (s, 1H), 7.69–7.57 (m, 3H), 7.48 (d, *J* = 8.1 Hz, 1H), 7.22 (d, *J* = 5.6 Hz, 3H), 7.18 (t, *J* = 7.6 Hz, 1H), 7.05 (t, *J* = 7.4 Hz, 1H); ^13^C-NMR (101 MHz, DMSO) δ 146.6, 137.1, 129.1, 128.3, 123.3, 121.3, 120.2, 112.4, 102.1.

*2-(1H-Indol-2-yl)-1H-benzo[d]imidazole* (**AI-25**). Yellow solid; yield 71%; ^1^H-NMR (400 MHz, DMSO-*d*_6_) δ 12.14 (s, 1H), 9.00 (s, 1H), 8.32 (t, *J* = 23.5 Hz, 1H), 7.64 (dd, *J* = 22.2, 6.3 Hz, 2H), 7.52 (d, *J* = 8.0 Hz, 1H), 7.36 (s, 1H), 7.21 (t, *J* = 7.3 Hz, 1H), 7.06 (t, *J* = 7.2 Hz, 1H).

*3-(Ethylthio)-2-phenylimidazo[1,2-a]pyridine* (**AI-26**). Brown oil; yield 71%; ^1^H-NMR (400 MHz, CDCl_3_) δ 8.52 (d, *J* = 6.9 Hz, 1H), 8.32 (d, *J* = 7.3 Hz, 2H), 7.67 (d, *J* = 9.0 Hz, 1H), 7.47 (t, *J* = 7.6 Hz, 2H), 7.37 (t, *J* = 7.3 Hz, 1H), 7.31–7.26 (m, 1H), 6.91 (t, *J* = 6.5 Hz, 1H), 2.69 (q, *J* = 7.4 Hz, 2H), 1.11 (t, *J* = 7.4 Hz, 3H).

*3-Ethyl-2-phenylimidazo[1,2-a]pyridine* (**AI-27**). Yellow solid; yield 68%; ^1^H-NMR (400 MHz, CDCl_3_) δ 7.90–7.87 (m, 1H), 7.79–7.72 (m, 2H), 7.62 (d, *J* = 9.1 Hz, 1H), 7.43 (dd, *J* = 10.3, 5.1 Hz, 2H), 7.34–7.29 (m, 1H), 7.10 (ddd, *J* = 9.0, 6.7, 1.2 Hz, 1H), 6.75 (td, *J* = 6.8, 1.1 Hz, 1H), 3.03 (q, *J* = 7.5 Hz, 2H), 1.29 (dd, *J* = 9.9, 5.2 Hz, 3H).

*3-Nitro-2-phenylimidazo[1,2-a]pyridine* (**AI-28**). Yellowish-brown solid; yield 77%; ^1^H-NMR (400 MHz, CDCl_3_) δ 9.52 (d, *J* = 7.0 Hz, 1H), 7.93–7.87 (m, 2H), 7.85 (d, *J* = 8.9 Hz, 1H), 7.70–7.61 (m, 1H), 7.54–7.47 (m, 3H), 7.32–7.26 (m, 1H).

*2-Phenylquinazolin-4(3H)-one* (**AI-29**). White solid; yield 80%; ^1^H-NMR (400 MHz, DMSO-*d*_6_) δ 12.54 (s, 1H), 8.17 (t, *J* = 8.8 Hz, 3H), 7.87–7.67 (m, 2H), 7.53 (dd, *J* = 17.6, 8.2 Hz, 4H).

*2-(Thiazol-4-yl)quinazolin-4(3H)-one* (**AI-30**). Off-white solid; yield 62%; ^1^H-NMR (400 MHz, CDCl_3_) δ 10.38 (s, 1H), 8.90 (d, *J* = 2.1 Hz, 1H), 8.48 (d, *J* = 2.0 Hz, 1H), 8.33 (d, *J* = 7.9 Hz, 1H), 7.80–7.76 (m, 2H), 7.50 (ddd, *J* = 8.1, 5.2, 3.1 Hz, 1H).

*2-Phenyl-2H-indazole* (**AI-31**). Orange solid; yield 66%; ^1^H-NMR (400 MHz, CDCl_3_) δ 8.38 (s, 1H), 7.87 (dd, *J* = 8.6, 1.0 Hz, 2H), 7.79–7.73 (m, 1H), 7.68 (d, *J* = 8.5 Hz, 1H), 7.54–7.45 (m, 2H), 7.37 (t, *J* = 7.4 Hz, 1H), 7.28 (ddt, *J* = 11.6, 6.0, 3.0 Hz, 1H), 7.08 (dd, *J* = 8.1, 7.0 Hz, 1H).

*2-(2-Bromophenyl)imidazo[1,2-a]pyridine* (**AI-32**). Brown solid; yield 73%; ^1^H-NMR (400 MHz, CDCl_3_) δ 8.29 (s, 1H), 8.19–8.10 (m, 2H), 7.65 (dd, *J* = 13.3, 8.6 Hz, 2H), 7.41 (t, *J* = 7.5 Hz, 1H), 7.18 (t, *J* = 7.8 Hz, 2H), 6.79 (t, *J* = 6.7 Hz, 1H).

*2-(2-Bromophenyl)-7-methylimidazo[1,2-a]pyridine* (**AI-33**). Brown solid; yield 70%; ^1^H-NMR (400 MHz, CDCl_3_) δ 8.17(d, *J* = 7.5 Hz, 1H), 8.14 (dd, *J* = 7.8, 1.3 Hz, 1H), 7.98 (d, *J* = 6.9 Hz, 1H), 7.64 (d, *J* = 7.8 Hz, 1H), 7.42–7.32 (m, 2H), 7.19–7.09 (m, 1H), 6.58 (d, *J* = 6.9 Hz, 1H), 2.37 (s, 3H).

*5-Methoxy-2-phenylisoindoline-1,3-dione* (**AI-34**). Off-white solid; yield 81%; ^1^H-NMR (400 MHz, CDCl_3_) δ 7.86 (d, *J* = 8.3 Hz, 1H), 7.54–7.47 (m, 2H), 7.46–7.37 (m, 4H), 7.23 (dd, *J* = 8.3, 2.3 Hz, 1H), 3.96 (s, 3H).

### 3.2. Biological Evaluation

#### 3.2.1. General

All synthetic molecules were dissolved at a concentration of 30 mM in 100% DMSO as a stock solution, stored at −20 °C, and diluted with medium before each experiment. The final DMSO concentration did not exceed 0.1% throughout the study (all the control groups are composed of 0.1% DMSO). Lipopolysaccharide (LPS) and adenosine triphosphate (ATP) were purchased from Sigma-Aldich (St. Louis, MO, USA). ELISA kits for human IL-1β were purchased from Shanghai Yuanye BioTechnology Co., Ltd. (Shanghai, China).

Human THP-1 cells were purchased from Shanghai Institute of Cell Biology (Shanghai, China) and maintained in RPMI 1640 medium, supplemented with 100 U/mL of penicillin, 100 μg/mL of streptomycin and 10% fetal calf serum under a humidified 5% (*v*/*v*) CO_2_ atmosphere at 37 °C.

#### 3.2.2. IL-1β Release Inhibition Analysis by ELISA

THP-1 cells were maintained in RPMI 1640 medium, supplemented with 10% FBS under a humidified 5% (*v*/*v*) CO_2_ atmosphere at 37 °C.

THP-1 cells were cultured with 500 nM PMA for 3 h, 5 × 10^6^ cells were seeded into every well of 96-well plates and primed by 100 ng/mL LPS for 3 h, then treated with a series of compounds at a dose of 10 μM for 1 h, respectively, following by 5 mM ATP treatment for 1 h. Released IL-1β in the supernatant was analyzed by ELISA (Dakewe Biotech Co., Ltd., Shenzhen, China) following the manufacturer’s instructions.

To determine cell viability, we directly lysed the cells and then subjected them to Cell Titer-Glo Luminescent Cell Viability Assay (Promega Co., Ltd., Beijing, China) to measure the level of ATP present in live cells. The assay was also performed following the manufacturer’s instructions.

## 4. Conclusions

In conclusion, two series of benzimidazole derivatives and analogs were designed, synthesized, and their in vitro anti-inflammatory activities were evaluated. From the first series of compounds, we identified three compounds, **TBZ-09**, **TBZ-11** and **TBZ-21**, as lead compounds. We then synthesized 34 more derivatives and identified several anti-inflammatory agents more potent than Fc11a-2. Efforts for developing new chemical entities targeting NLRP3 inflamasome for the treatment of clinical chronic and acute inflammatory diseases are ongoing in our lab.

## Figures and Tables

**Table 1:** Structures of TBZ derivatives.

No.	R_1_	R_2_	R_3_	R_4_	R_5_	R_6_
**TBZ**	H	H	H	H	H	4-thiazole
**TBZ-01**	H	Me	H	H	H	4-thiazole
**TBZ-02**	Me	H	H	H	H	4-thiazole
**TBZ-03**	H	Me	Me	H	H	4-thiazole
**TBZ-04**	H	H	Cl	H	H	4-thiazole
**TBZ-05**	H	H	F	H	H	4-thiazole
**TBZ-06 ^a^**	H	H	OMe	H	H	4-thiazole
**TBZ-07**	H	Bz	H	H	H	4-thiazole
**TBZ-08**	H	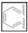		H	H	4-thiazole
**TBZ-09**	H	H	NO_2_	H	H	4-thiazole
**TBZ-10**	H	H	H	H	Me	4-thiazole
**TBZ-11**	H	H	H	H	Bn ^b^	4-thiazole
**TBZ-12**	H	H	H	H	pMB ^b^	4-thiazole
**TBZ-13**	H	H	H	H	mNB ^b^	4-thiazole
**TBZ-14**	Me	H	H	H	H	2-Py ^b^
**TBZ-15**	H	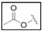	H	H	H	4-thiazole
**TBZ-16**	H	H	H	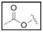	H	4-thiazole
**TBZ-17**	H	H	CF_3_	H	H	4-thiazole
**TBZ-18**	H	H	H	H	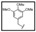	4-thiazole
**TBZ-19**	H	H	H	H	Bn ^b^	Bn ^b^
**TBZ-20**	H	H	H	H	2-Py ^b^	2-Py ^b^

^a^ Tautomer was determined by NMR spectroscopy analysis; ^b^ Bn= benzyl; *p*MB = *p*-methoxyl benzyl; *m*-NB = *m*-nitro benzyl; 2-Py = 2-pyridyl; Bz = benzoyl.

**Table 2:**
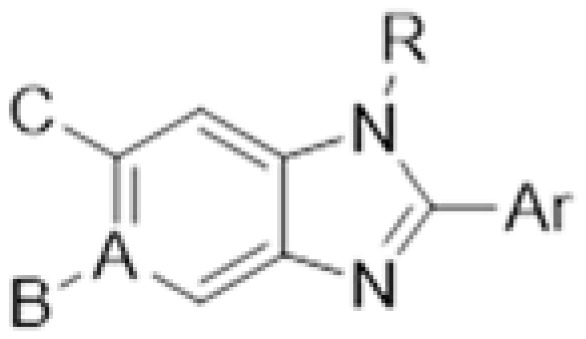
Structures of compounds **AI-1**–**25**.

No.	A	B	Ar	R	C
**AI-1**	N	-	phenyl	H	-
**AI-2**	N	-	4-flurophenyl	H	-
**AI-3**	N	-	3,4-methylendioxyphenyl	H	-
**AI-4**	N	-	3,4,-dimethoxylphenyl	H	-
**AI-5**	N	-	3,5-diflurophenyl	H	-
**AI-6**	N	-	4-*N*,*N*-dimethylphenyl	H	-
**AI-7**	N	-	2-fural	H	-
**AI-8**	N	-	3-indoyl	H	-
**AI-9**	N	-	2-indoyl	H	-
**AI-10**	C	NO_2_	phenyl	H	-
**AI-11**	C	NO_2_	4-flurophenyl	H	-
**AI-12**	C	NO_2_	3,4-methylendioxyphenyl	H	-
**AI-13**	C	NO_2_	3,4,-dimethoxylphenyl	H	-
**AI-14**	C	NO_2_	3,5-diflurophenyl	H	-
**AI-15**	C	NO_2_	4-*N*,*N*-dimethylphenyl	H	-
**AI-16**	C	NO_2_	2-fural	H	-
**AI-17**	C	NO_2_	3-indoyl	H	-
**AI-18**	C	H	4-thiazole	ethyl	-
**AI-19**	N	-	4-thiazole	allyl	-
**AI-20**	N	-	phenyl	ethyl	-
**AI-21**	N	-	4-thiazole	ethyl	-
**AI-22**	C	NO_2_	4-thiazole	benzyl	-
**AI-23**	C	H	4-thiazole	benzyl	NO_2_
**AI-24**	C	H	3-indoyl	H	-
**AI-25**	C	H	2-indoyl	H	-

## Supplementary Materials

Supplementary Materials can be accessed at: http://www.mdpi.com/1420-3049/22/2/213/s1. Supplementary materials containing spectral characterization details of **ALL** compounds are uploaded along with the manuscript and graphic files in PDF format.
