# Supplementary material for: Synthesis and Biological Evaluation of Novel Benzimidazole Derivatives and Analogs Targeting the NLRP3 Inflammasome
Source: Molecules. 2017 Jan 30;22(2):213. doi: 10.3390/molecules22020213 (PMC6155809; doi:10.3390/molecules22020213)
Supplement: Supplementary file 1 [file molecules-22-00213-s001.pdf]

Supplementary Materials: Synthesis and Biological Evaluation of Novel Benzimidazole Derivatives and Analogs Targeting the NLRP3 Inflammasome

Liangkun Pan, Nan Hang, Chao Zhang, Yu Chen, Shuchun Li, Yang Sun, Zhongjun Li and Xiangbao Meng

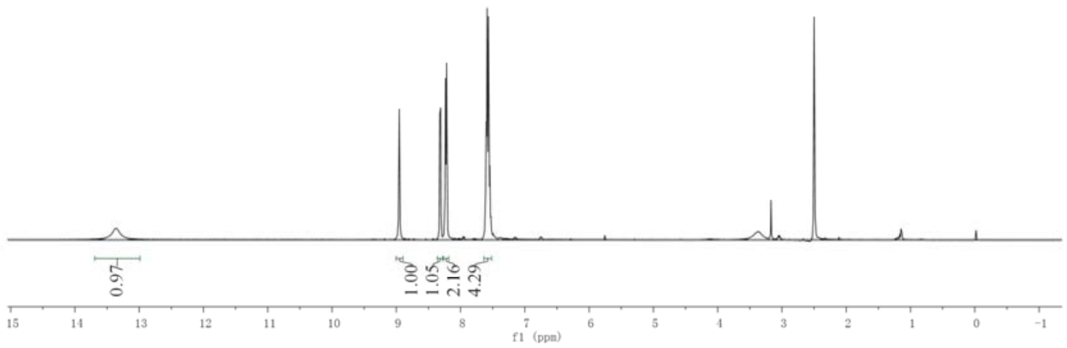

Figure S1. ^{1}H-NMR of AI-1.

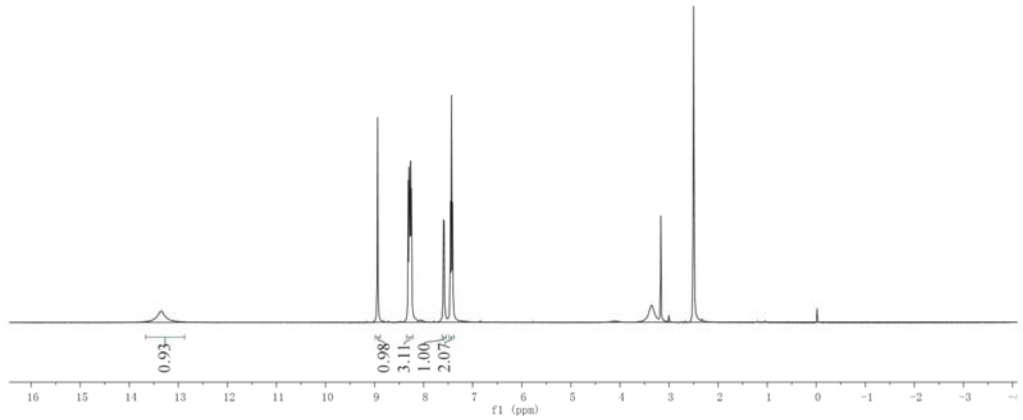

Figure S2. ^{1}H-NMR of AI-2.

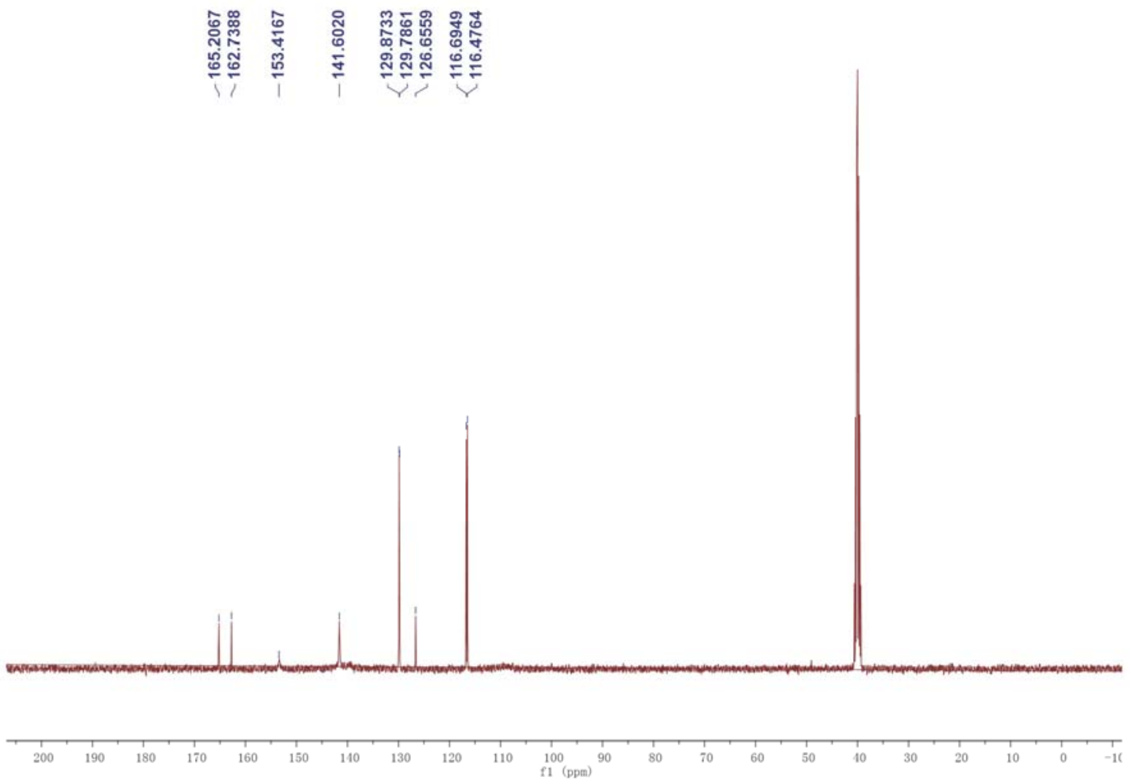

Figure S3. ^{13}C-NMR of AI-2.

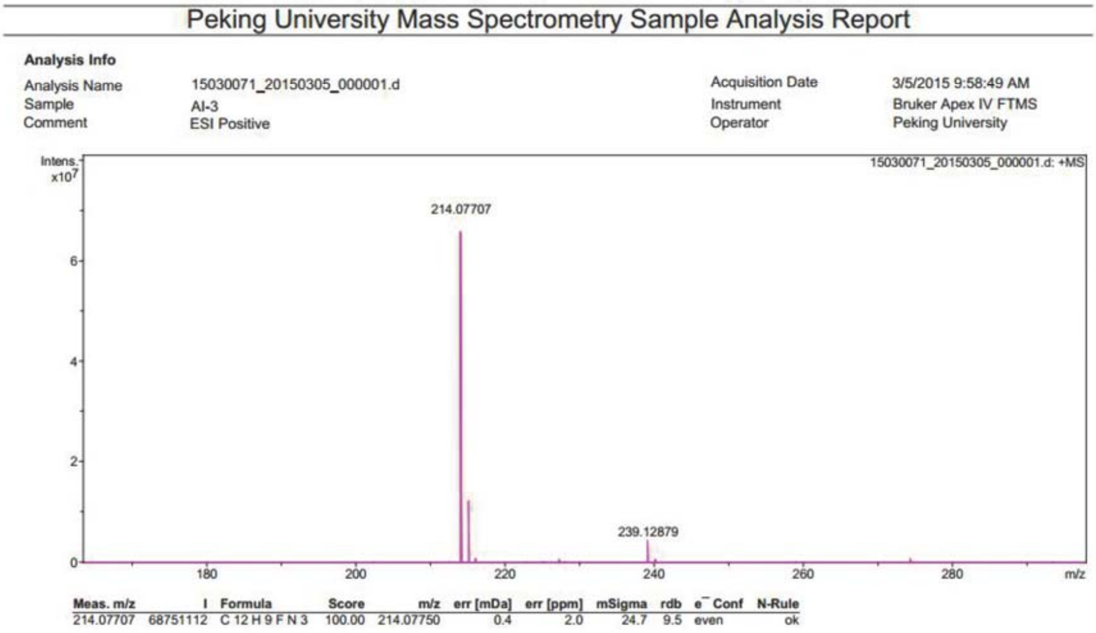

Figure S4. HR-EI-MS spectra of AI-2.

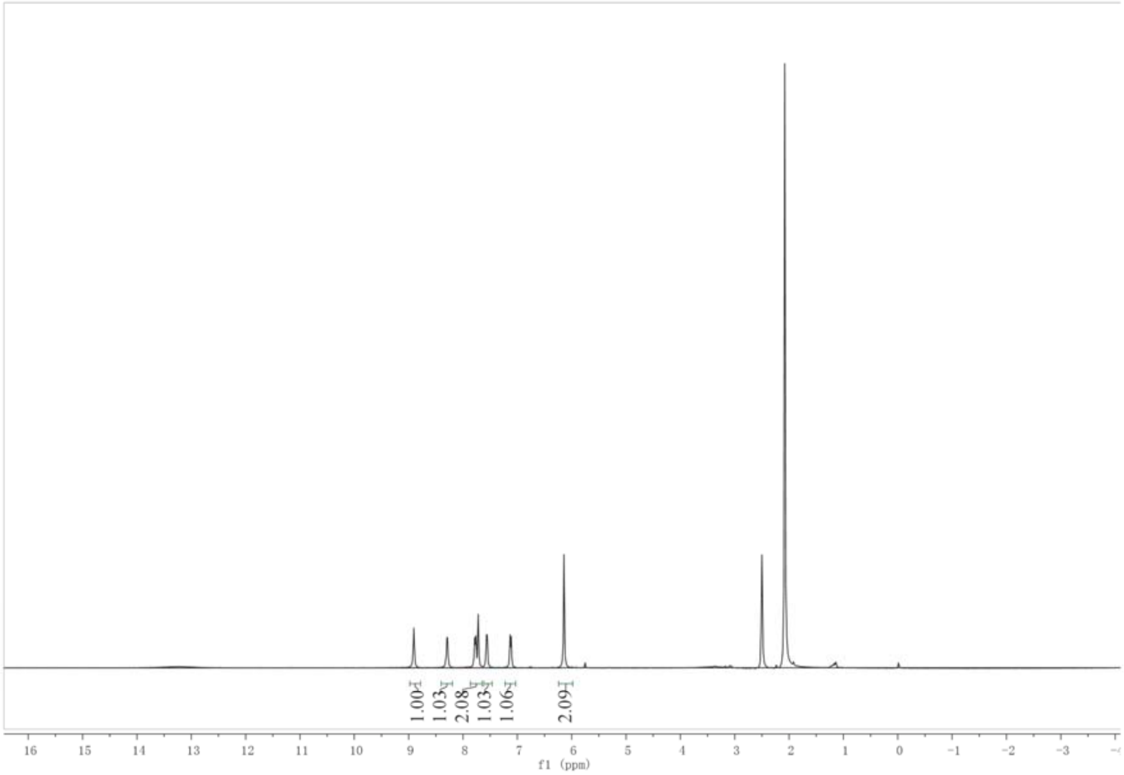

Figure S5. ^{1}H-NMR of AI-3.

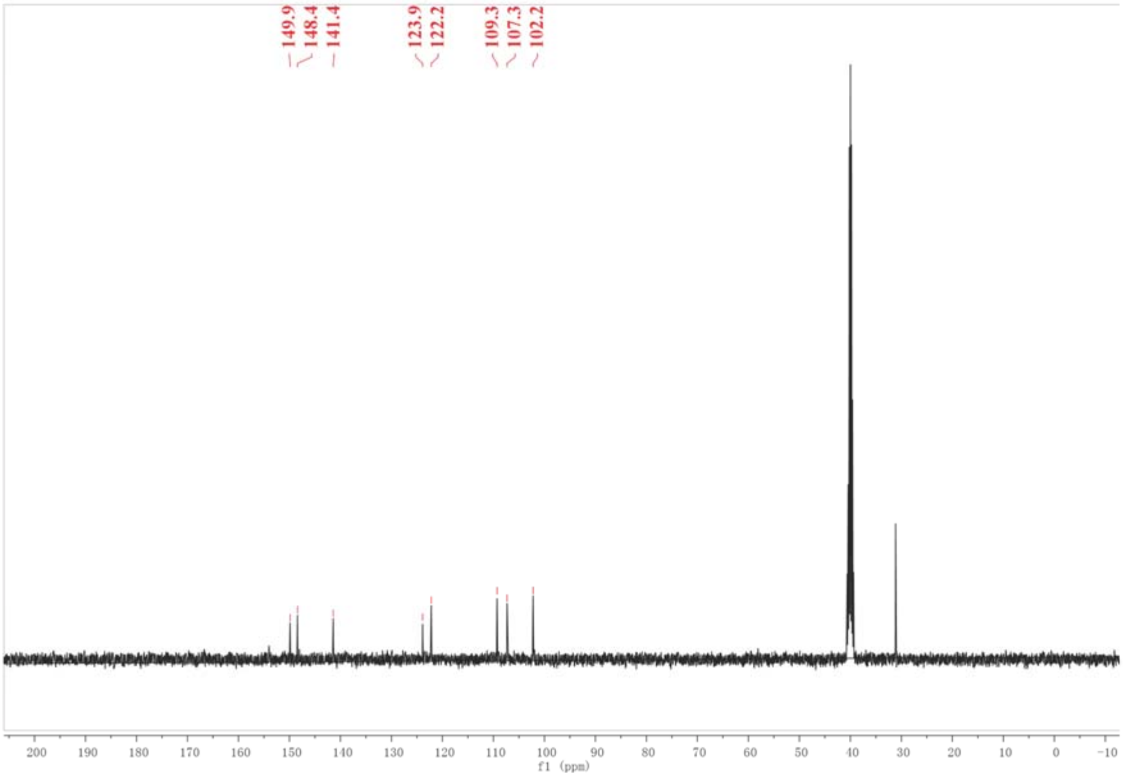

Figure S6. ^{13}C-NMR of AI-3.

Peking University Mass Spectrometry Sample Analysis Report

Analysis Info

Analysis Name	15030071_20150305_000001.d	Acquisition Date	3/5/2015 9:58:49 AM
Sample	AI-3	Instrument	Bruker Apex IV FTMS
Comment	ESI Positive	Operator	Peking University

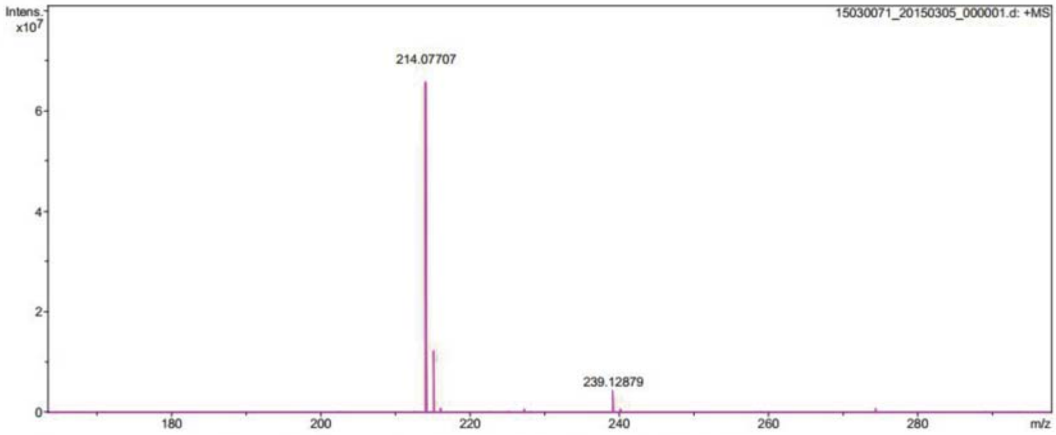

Meas. m/z	I	Formula	Score	m/z	err [mDa]	err [ppm]	mSigma	rdb	e⁻ Conf	N-Rule
214.07707	68751112	C 12 H 9 F N 3	100.00	214.07750	0.4	2.0	24.7	9.5	even	ok

Figure S7. HR-EI-MS spectra of AI-3.

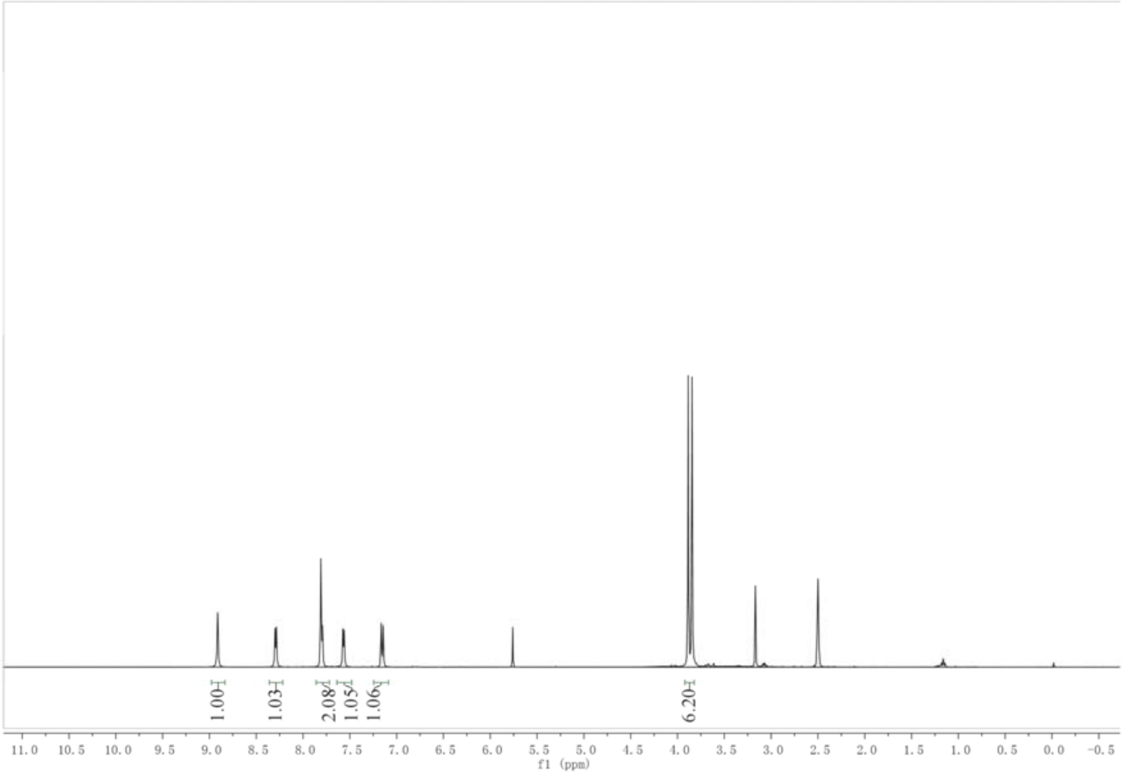

Figure S8. ^{1}H-NMR of AI-4.

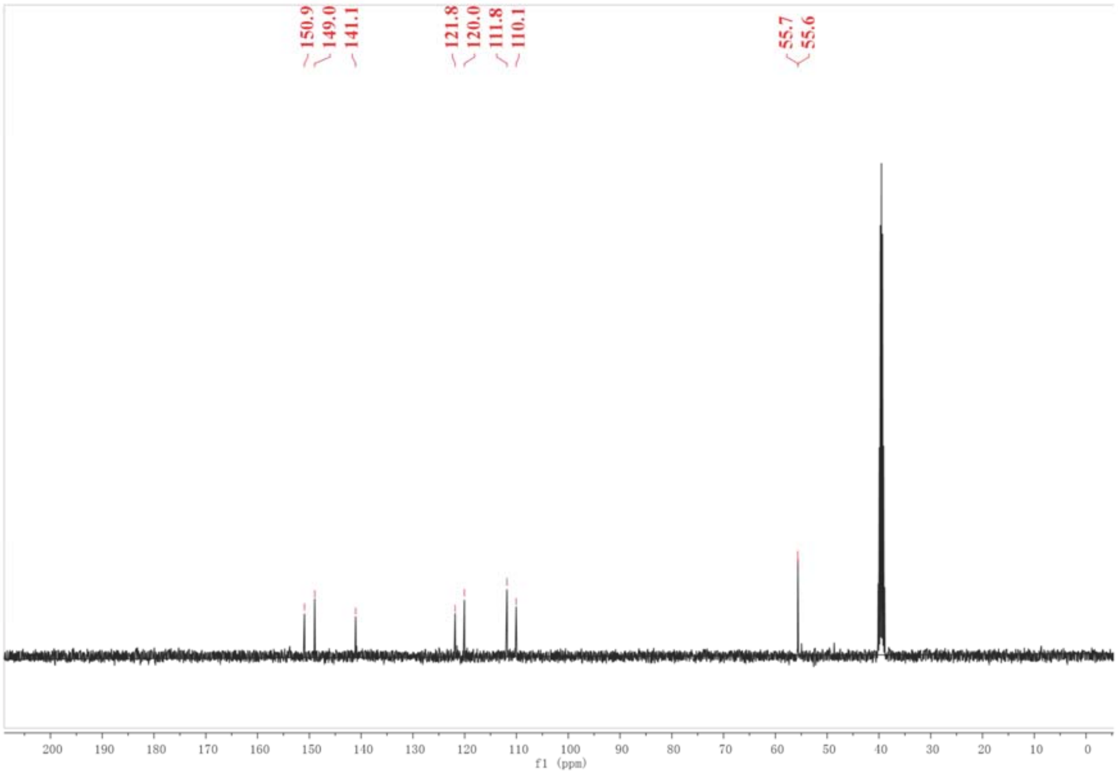

Figure S9. ^{13}C-NMR of AI-4.

Peking University Mass Spectrometry Sample Analysis Report

Analysis Info

Analysis Name	15030071_20150305_000003.d	Acquisition Date	3/5/2015 10:02:38 AM
Sample	AI-5	Instrument	Bruker Apex IV FTMS
Comment	ESI Positive	Operator	Peking University

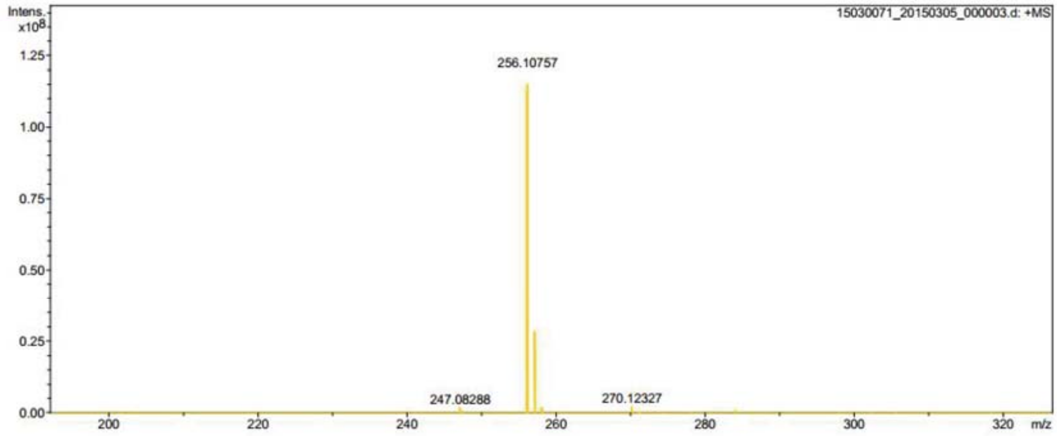

Meas. m/z	I	Formula	Score	m/z	err [mDa]	err [ppm]	mSigma	rdb	e⁻ Conf	N-Rule
256.10757	119631016	C 14 H 14 N 3 O 2	100.00	256.10805	0.5	1.9	47.5	9.5	even	ok

Figure S10. HR-EI-MS spectra of AI-4.

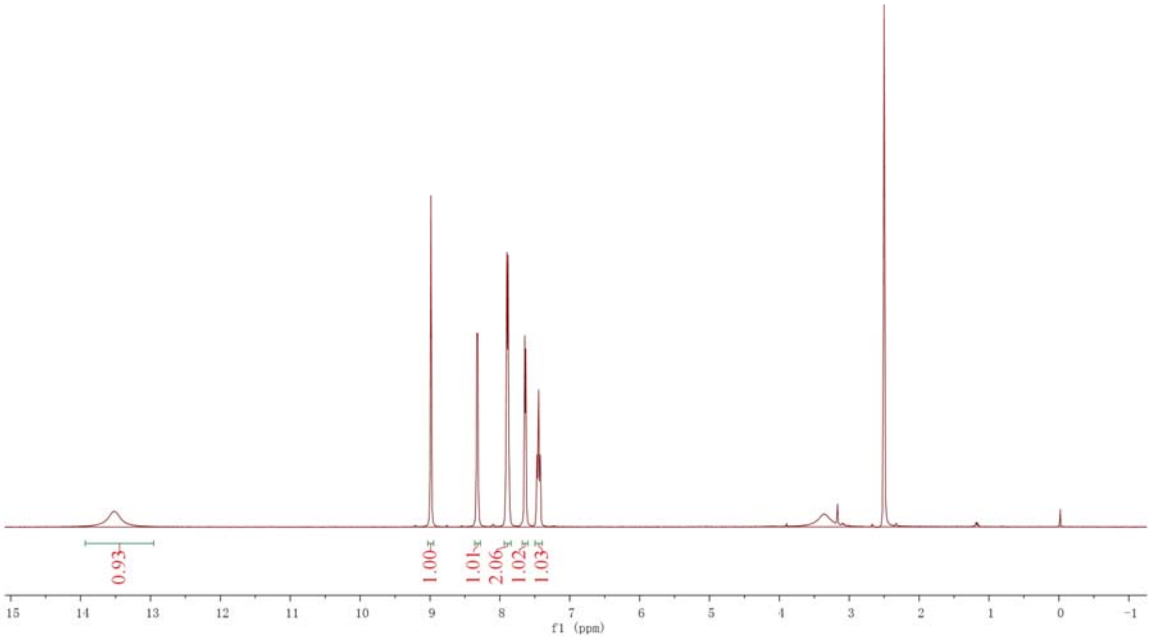

Figure S11. ^{1}H-NMR of AI-5.

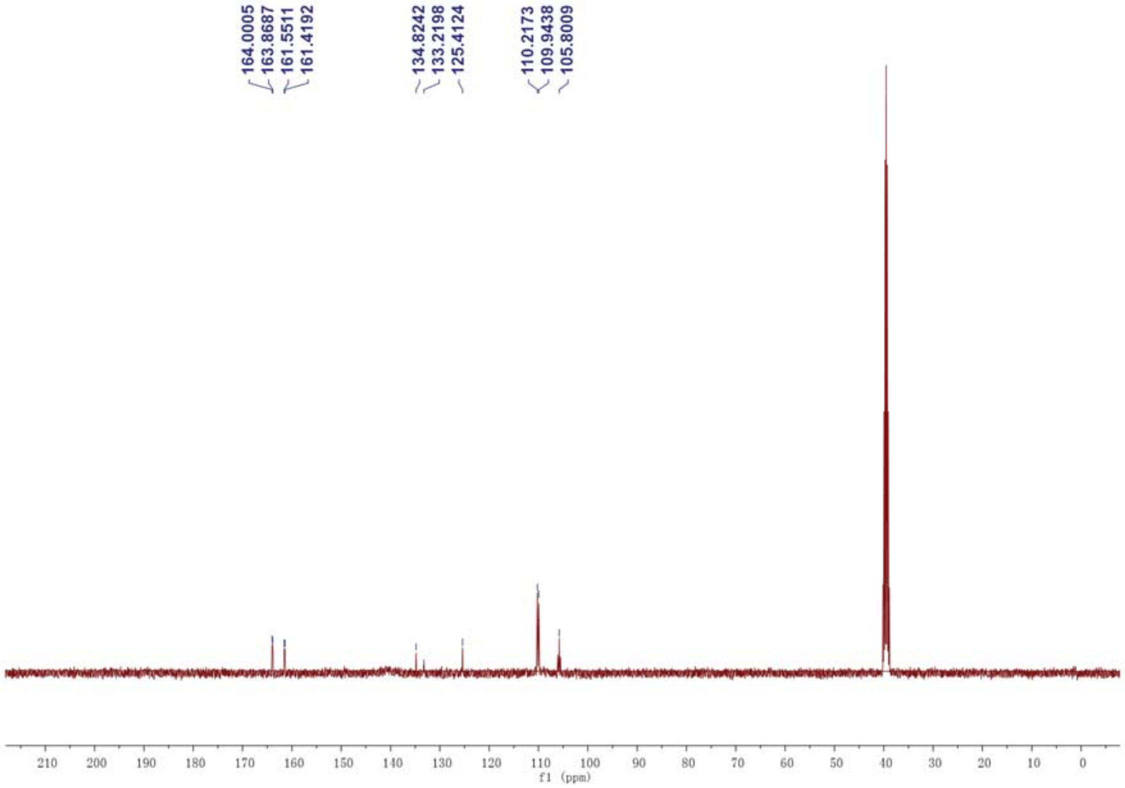

Figure S12. ^{13}C-NMR of AI-5.

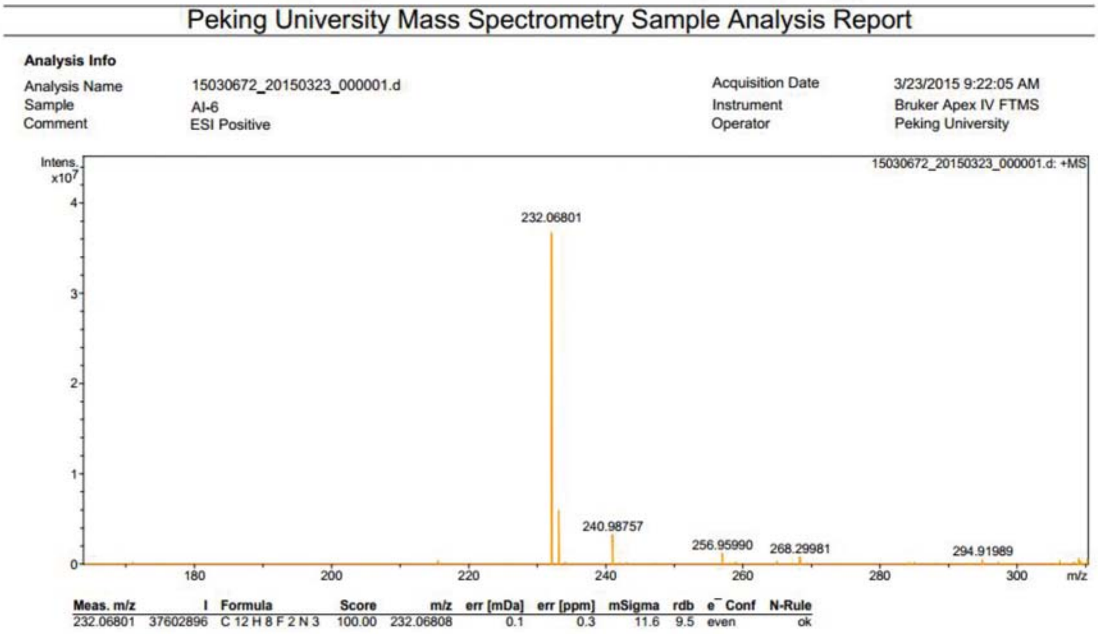

Figure S13. HR-EI-MS spectra of AI-5.

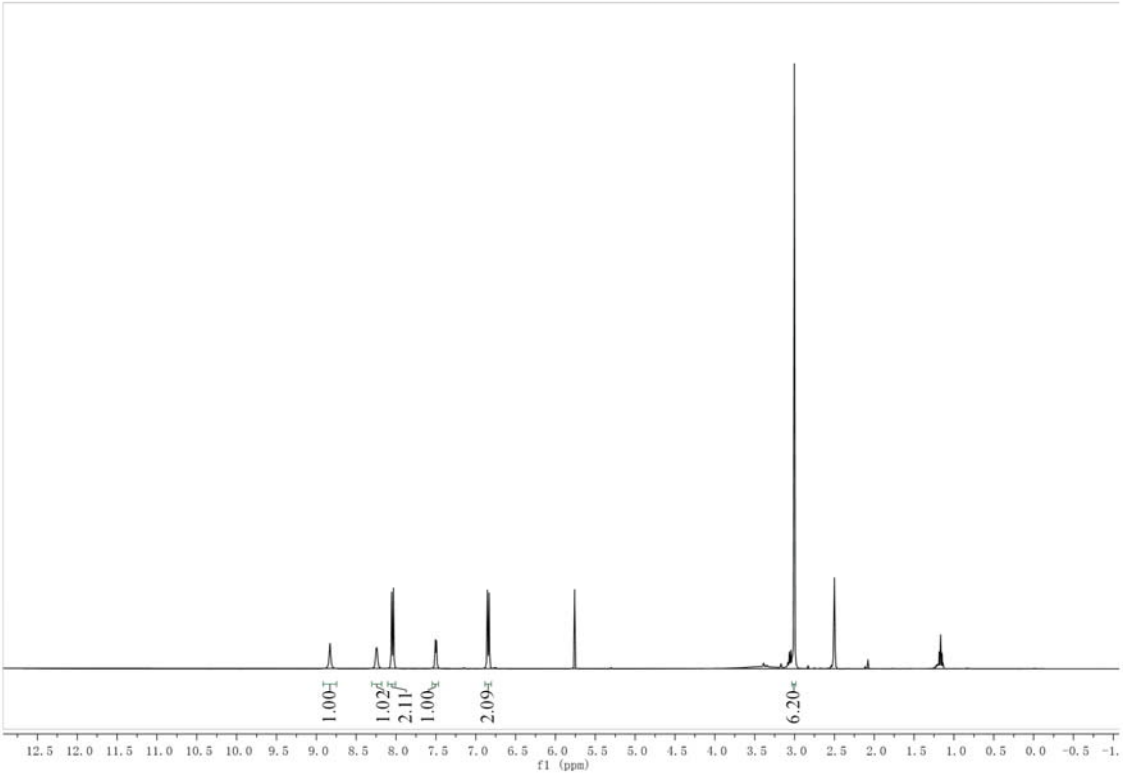

Figure S14. ^{1}H-NMR of AI-6.

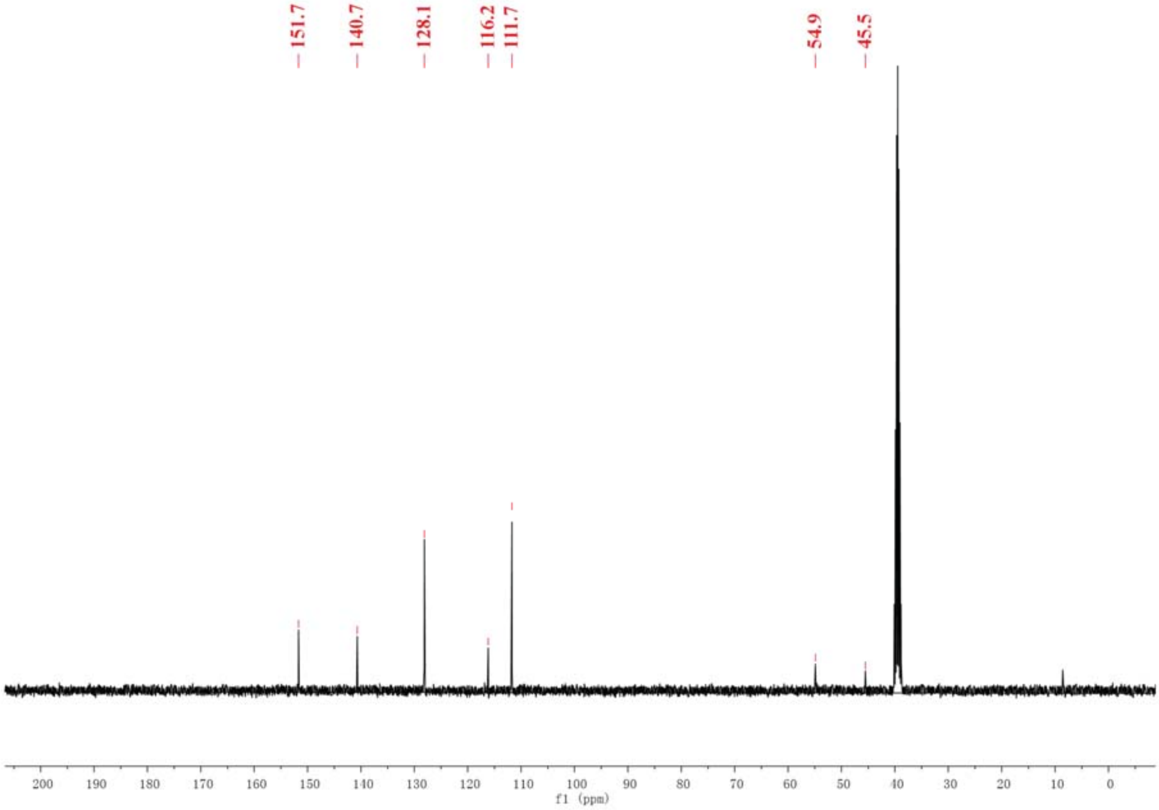

Figure S15. ^{13}C-NMR of AI-6.

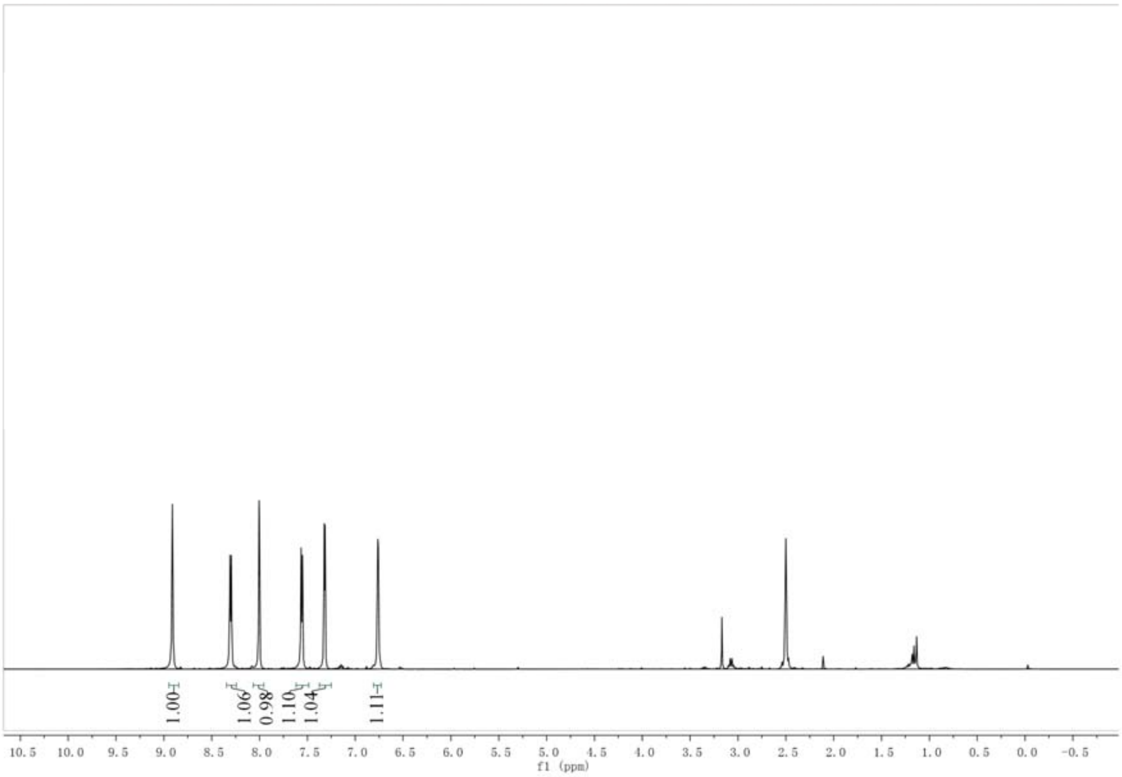

Figure S16. ^{1}H-NMR of AI-7.

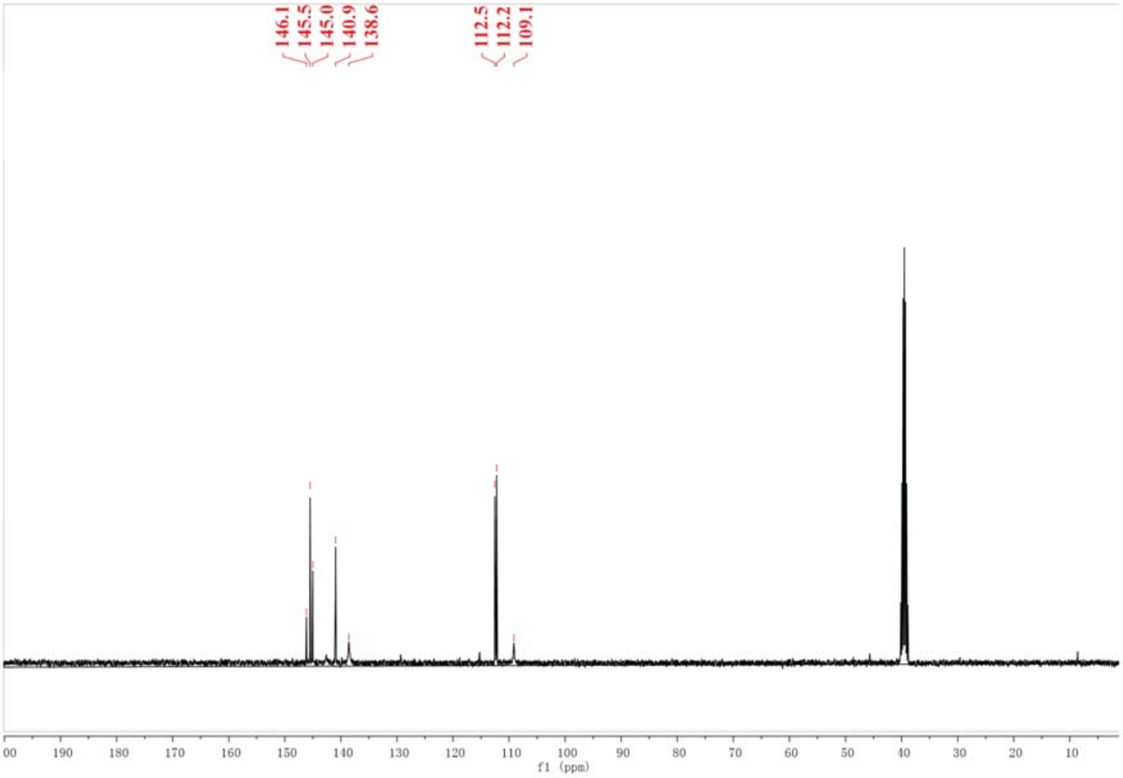

Figure S17. ^{13}C-NMR of AI-7.

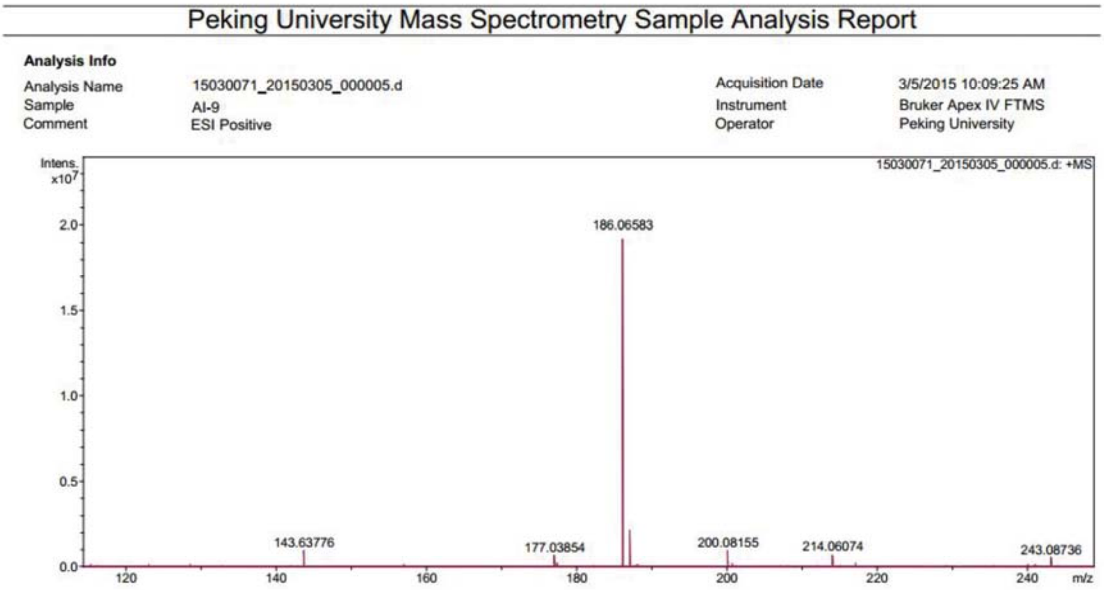

Meas. m/z	I	Formula	Score	m/z	err [mDa]	err [ppm]	mSigma	rdb	e⁻ Conf	N-Rule
186.06583	19528486	C 10 H 8 N 3 O	100.00	186.06619	0.4	1.9	3.2	8.5	even	ok

Figure S18. HR-EI-MS spectra of AI-7.

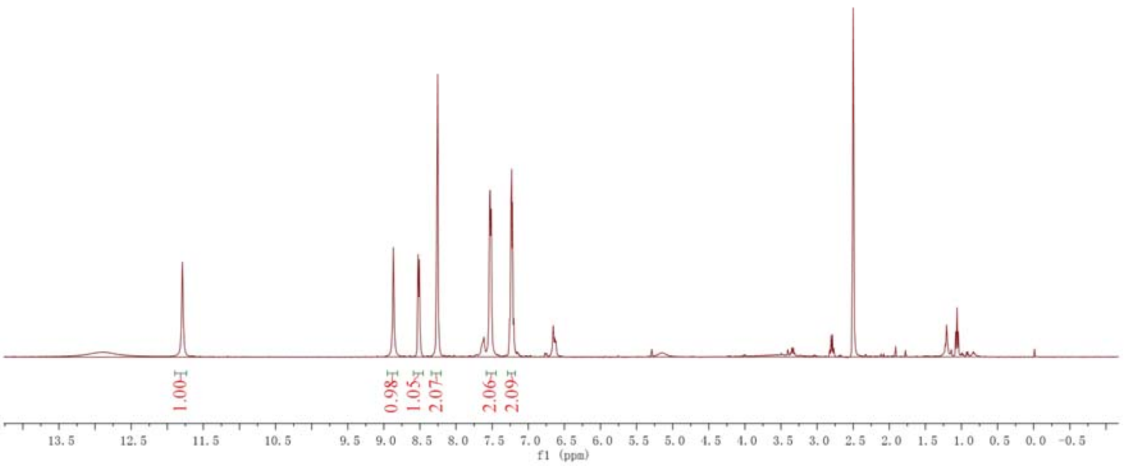

Figure S19. ^{1}H-NMR of AI-8.

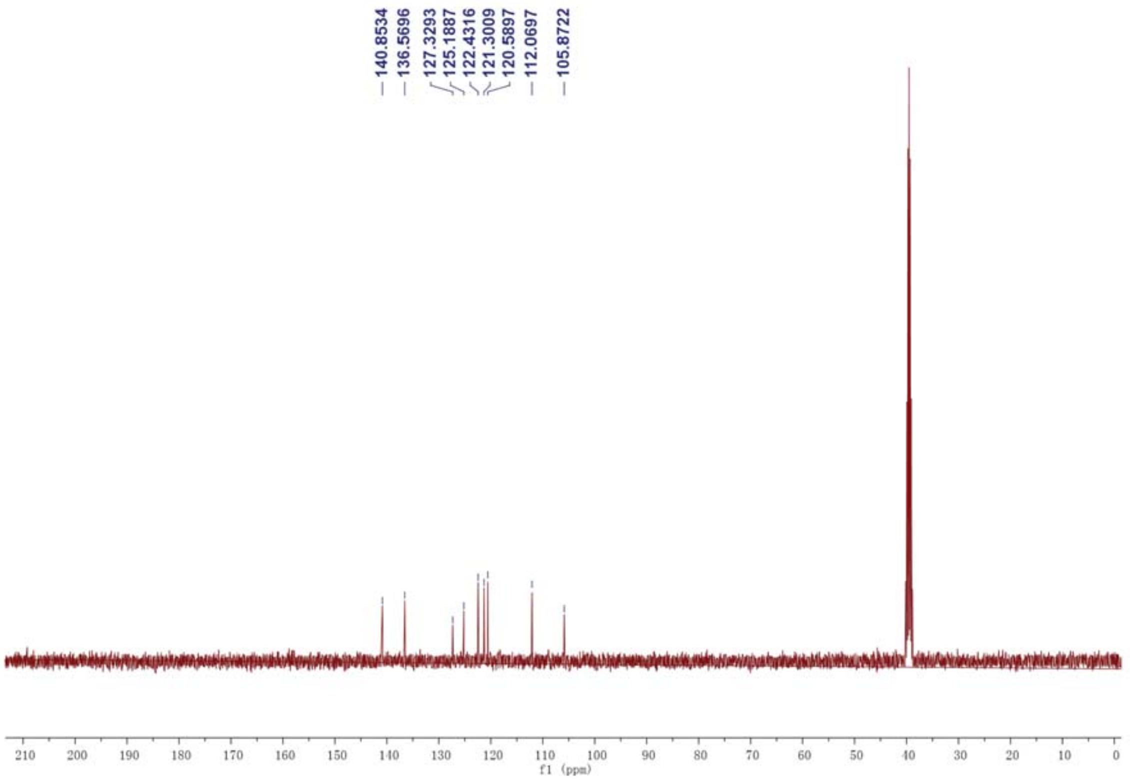

Figure S20. ^{13}C-NMR of AI-8.

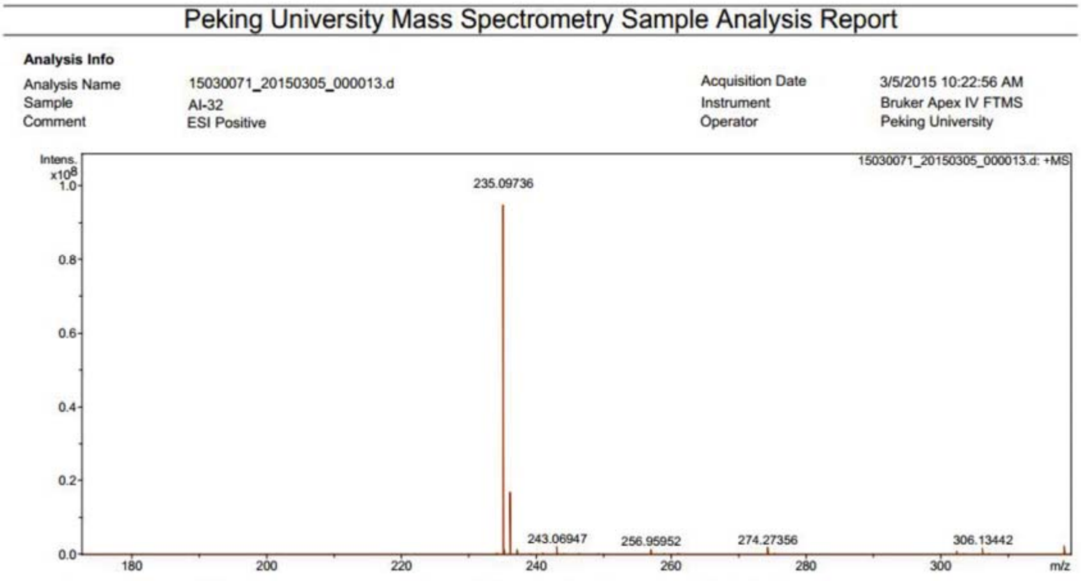

Meas. m/z	I	Formula	Score	m/z	err [mDa]	err [ppm]	mSigma	rdb	e⁻ Conf	N-Rule
235.09736	98481448	C 14 H 11 N 4	100.00	235.09782	0.5	2.0	5.3	11.5	even	ok

Figure S21. HR-EI-MS spectra of AI-8.

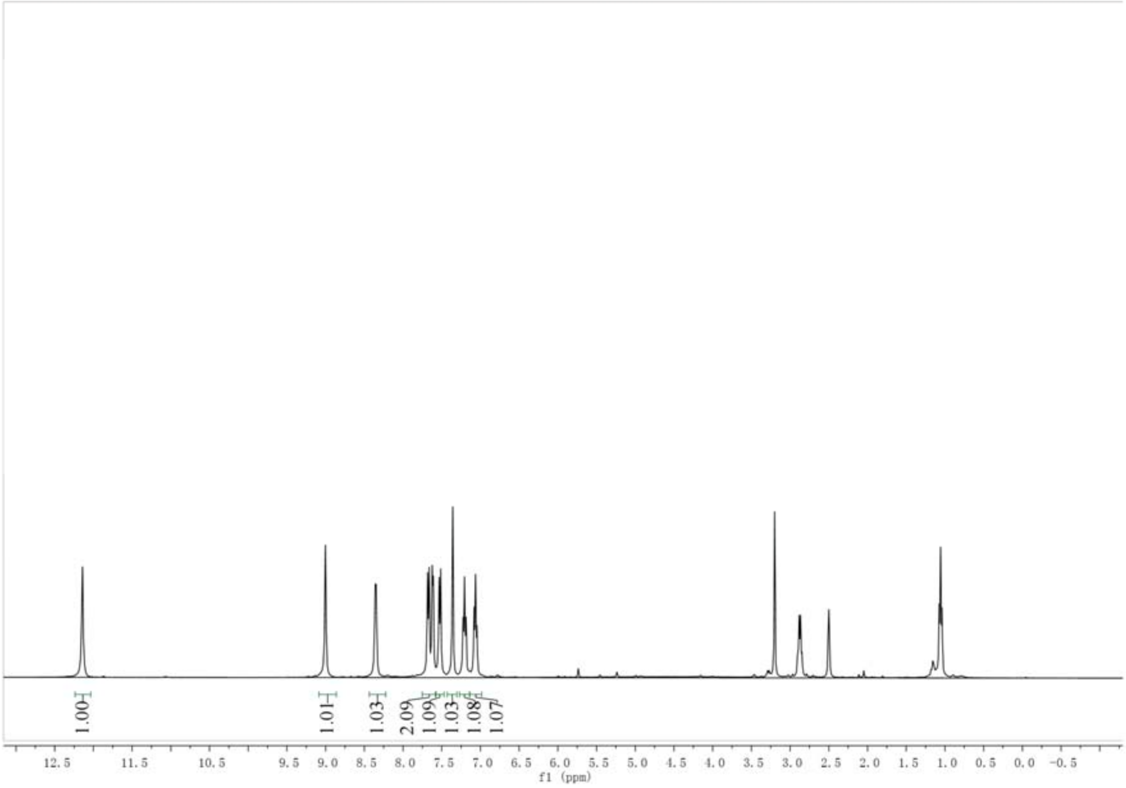

Figure S22. ^{1}H-NMR of AI-9.

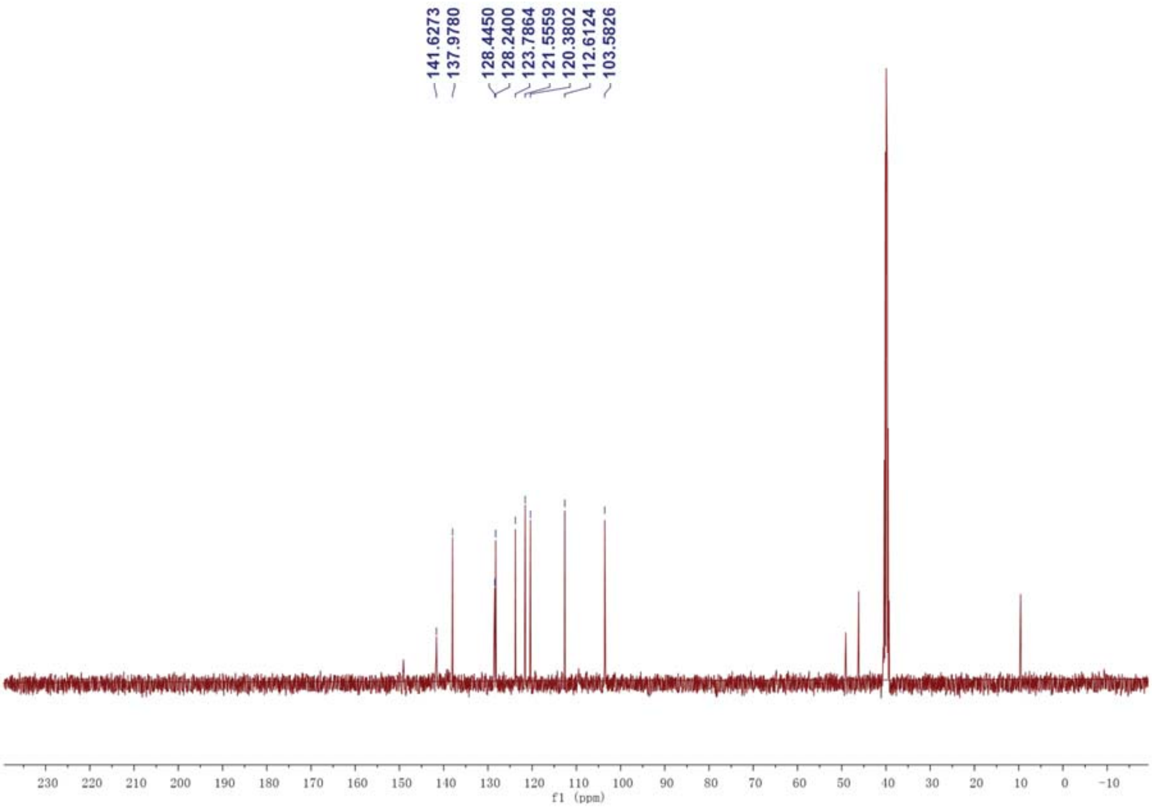

Figure S23. ^{13}C-NMR of AI-9.

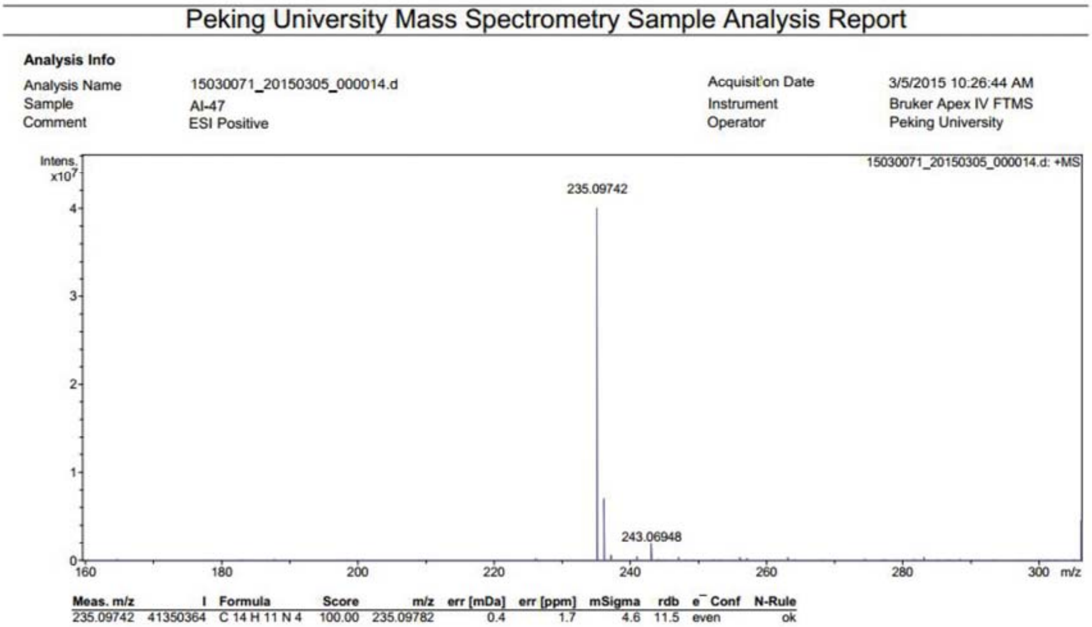

Figure S24. HR-EI-MS spectra of AI-9.

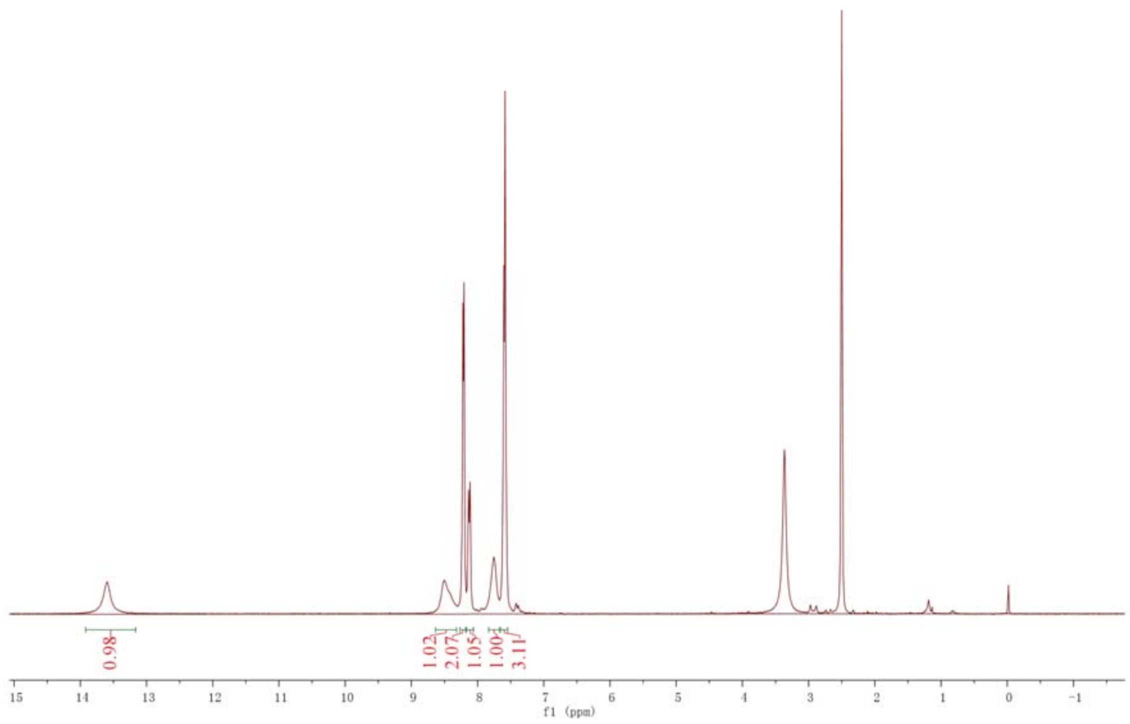

Figure S25. ^{1}H-NMR of AI-10.

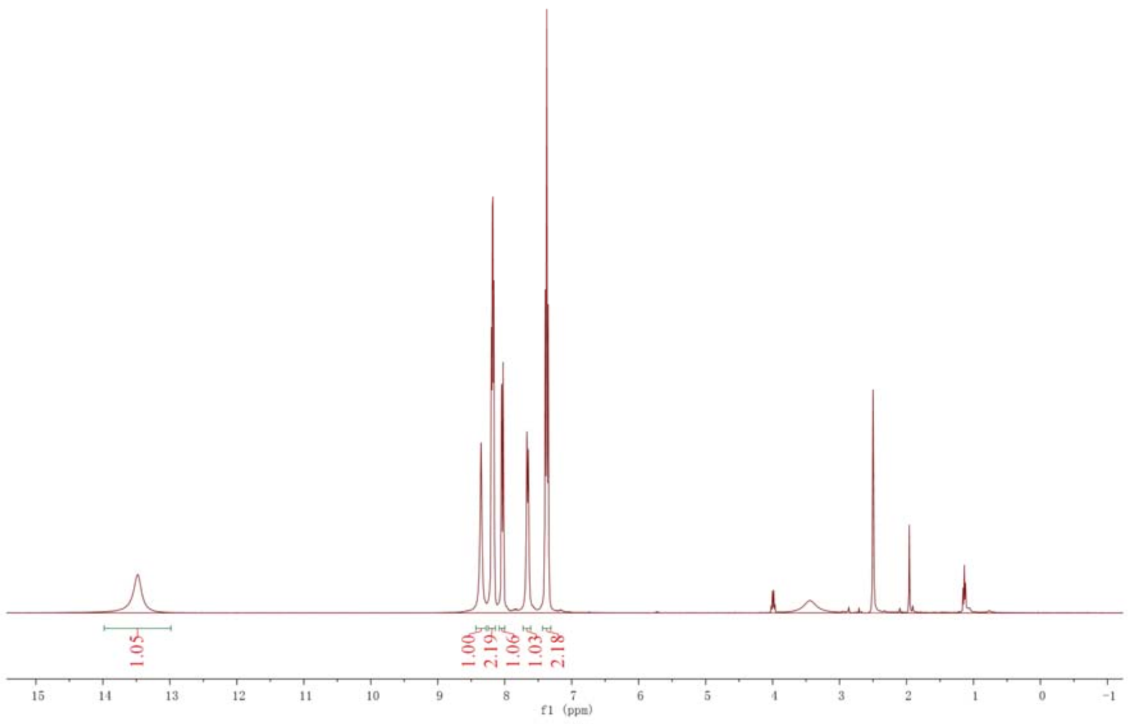

Figure S26. ^{1}H-NMR of AI-11.

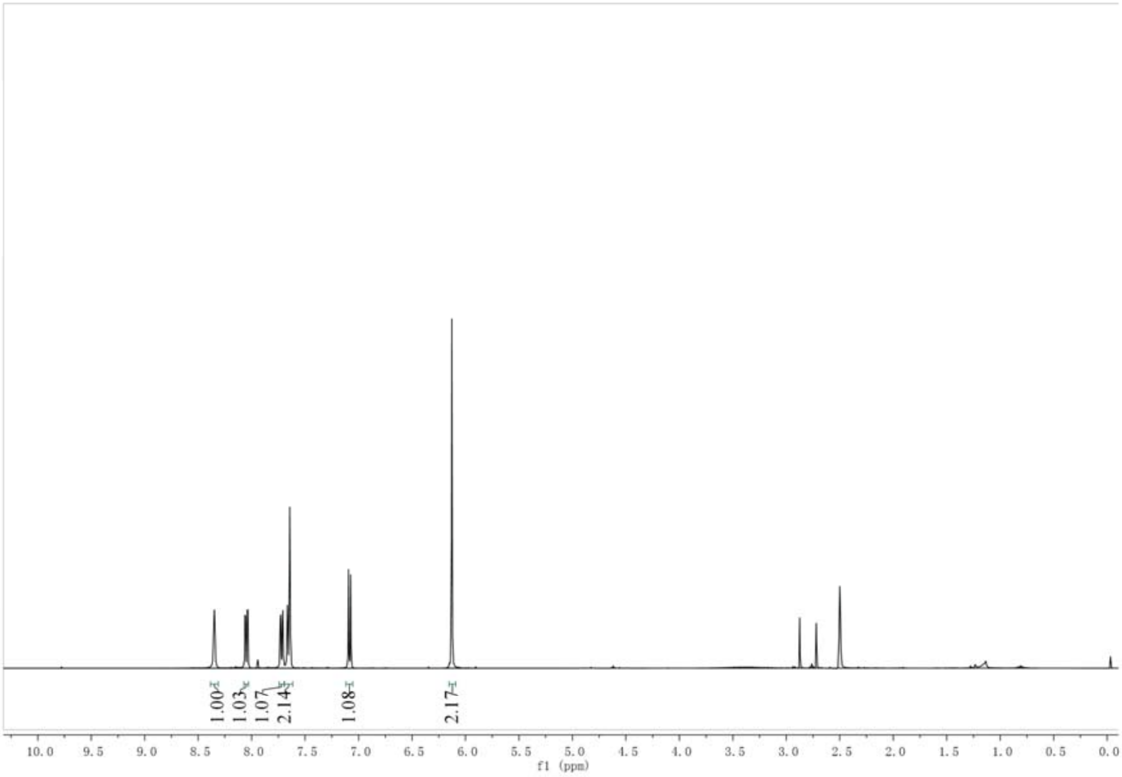

Figure S27. ^{1}H-NMR of AI-12.

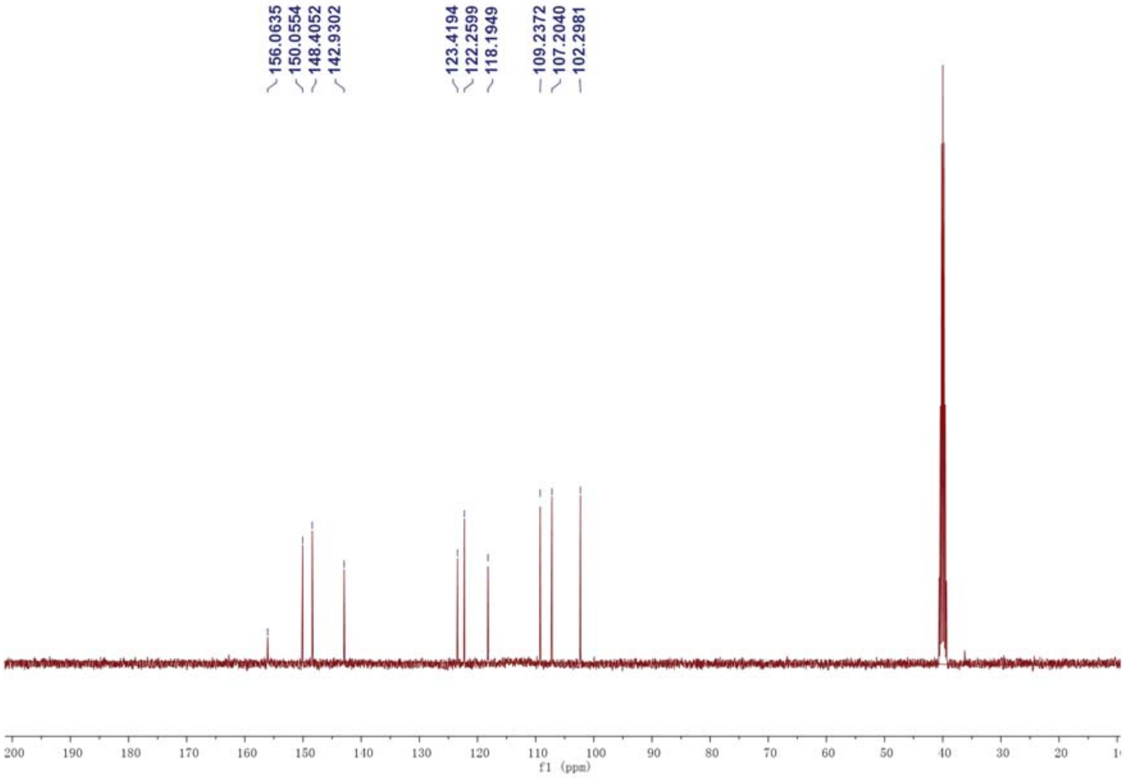

Figure S28. ^{13}C-NMR of AI-12.

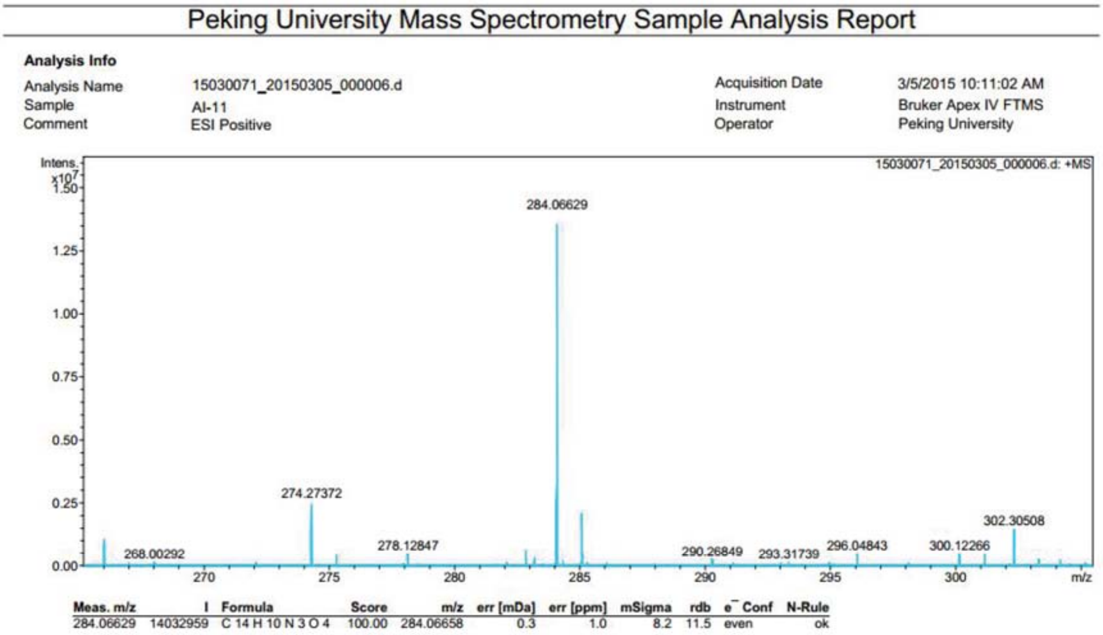

Figure S29. HR-EI-MS spectra of AI-12.

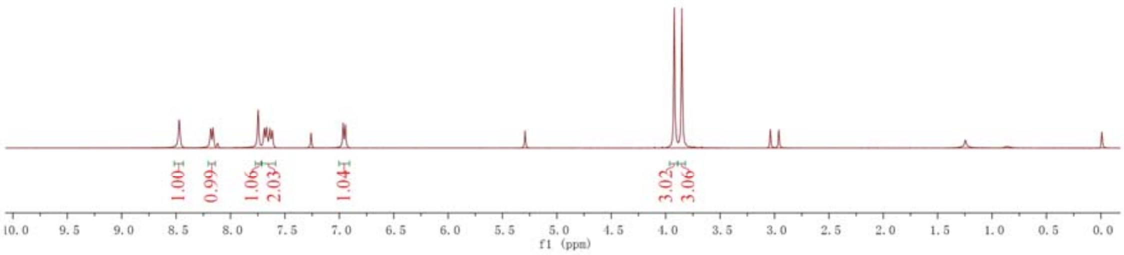

Figure S30. ^{1}H-NMR of AI-13.

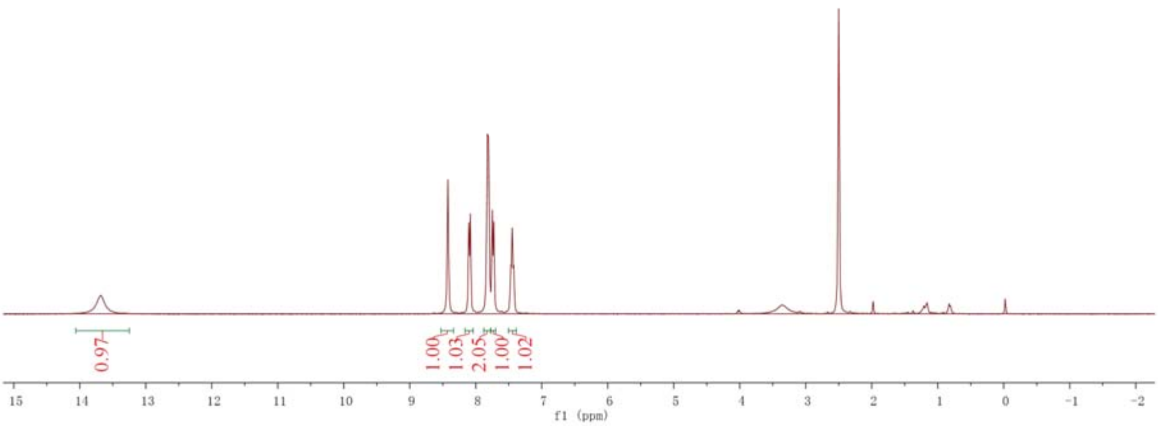

Figure S31. ^{1}H-NMR of AI-14.

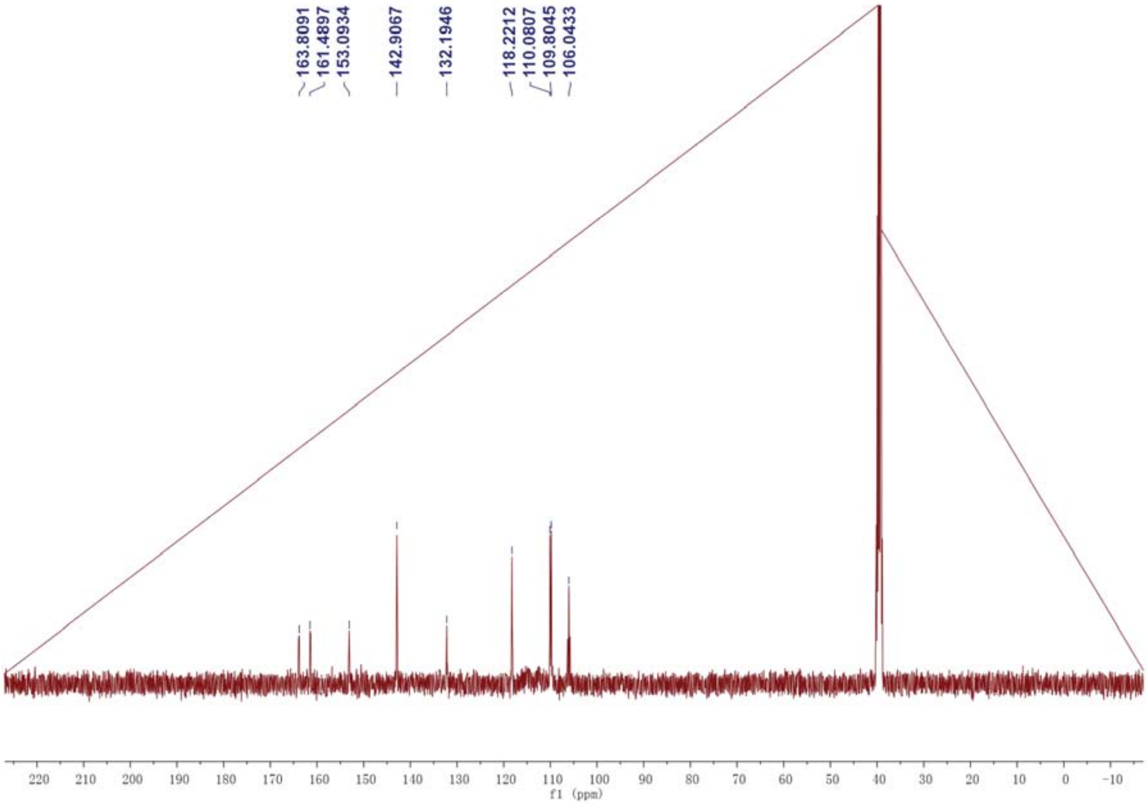

Figure S32. ^{13}C-NMR of AI-14.

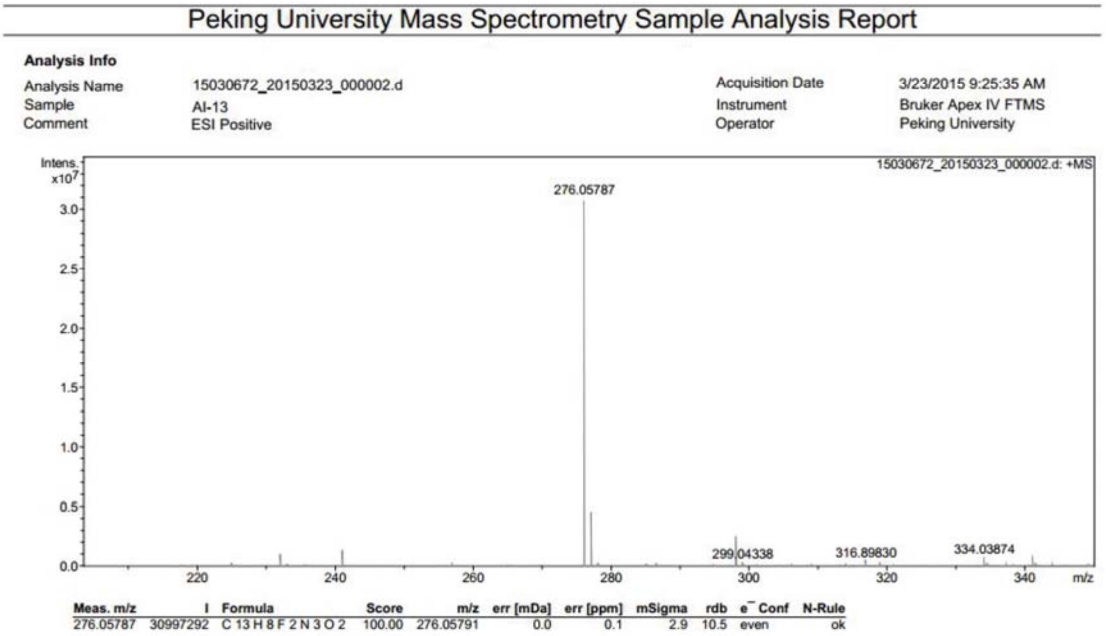

Figure S33. HR-EI-MS spectra of AI-14.

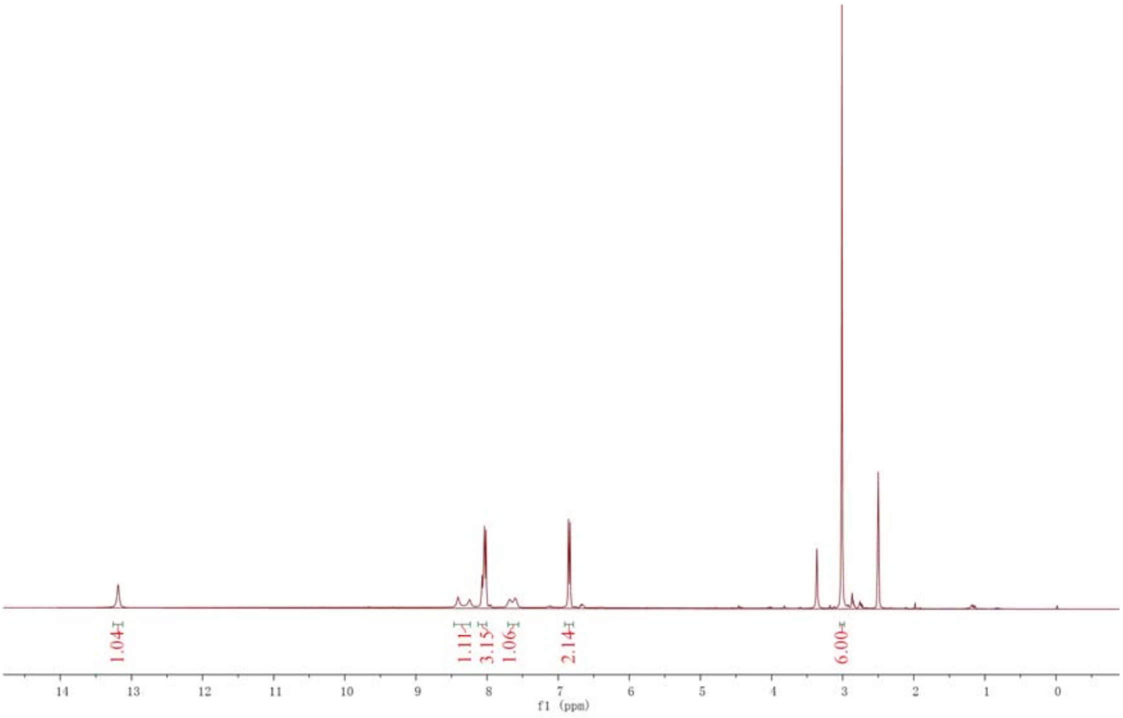

Figure S34. ^{1}H-NMR of AI-15.

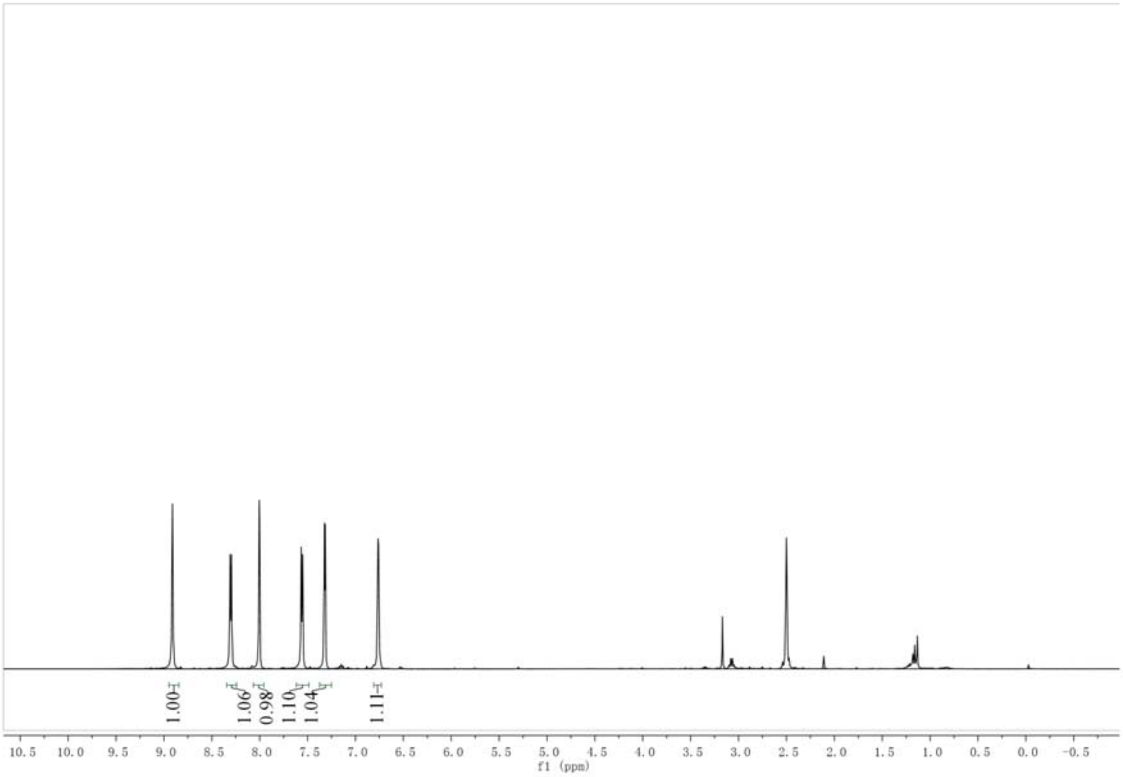

Figure S35. ^{1}H-NMR of AI-16.

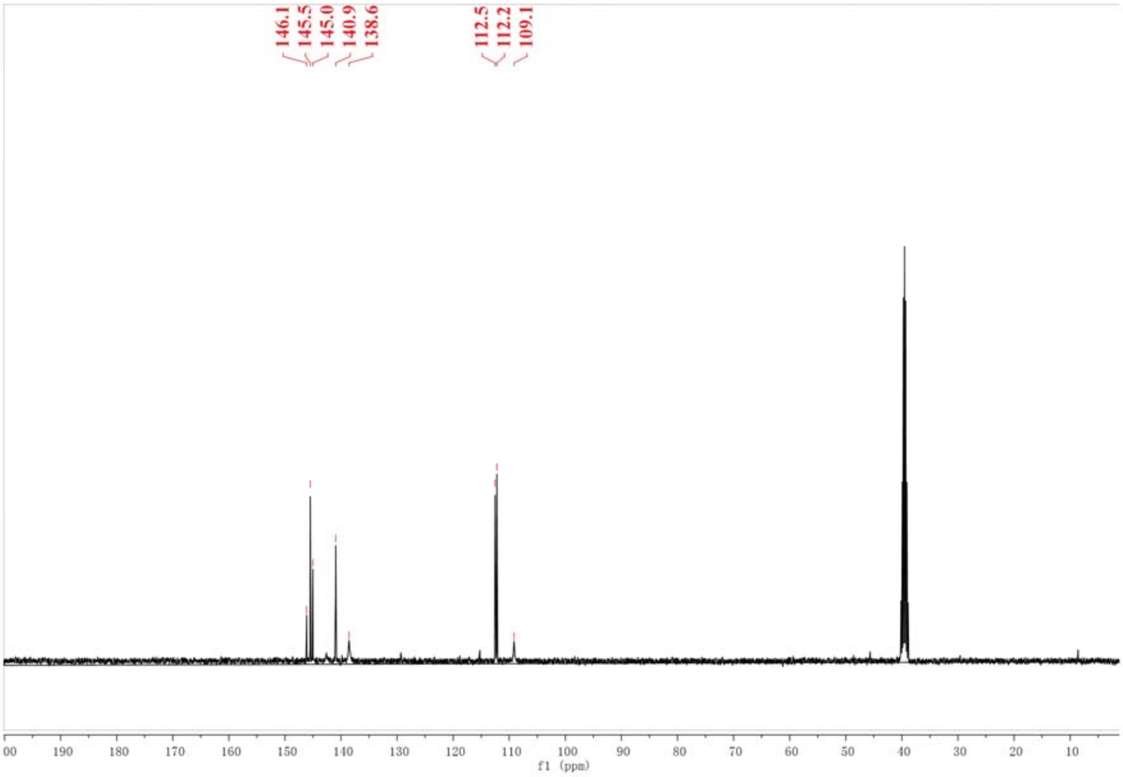

Figure S36. ^{13}C-NMR of AI-16.

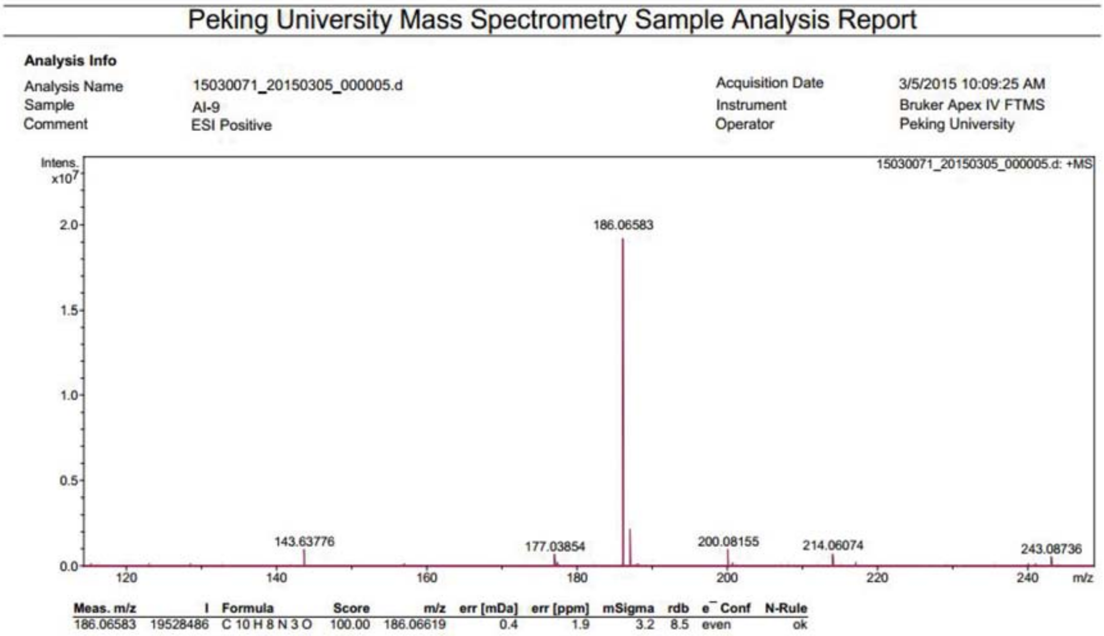

Figure S37. HR-EI-MS spectra of AI-16.

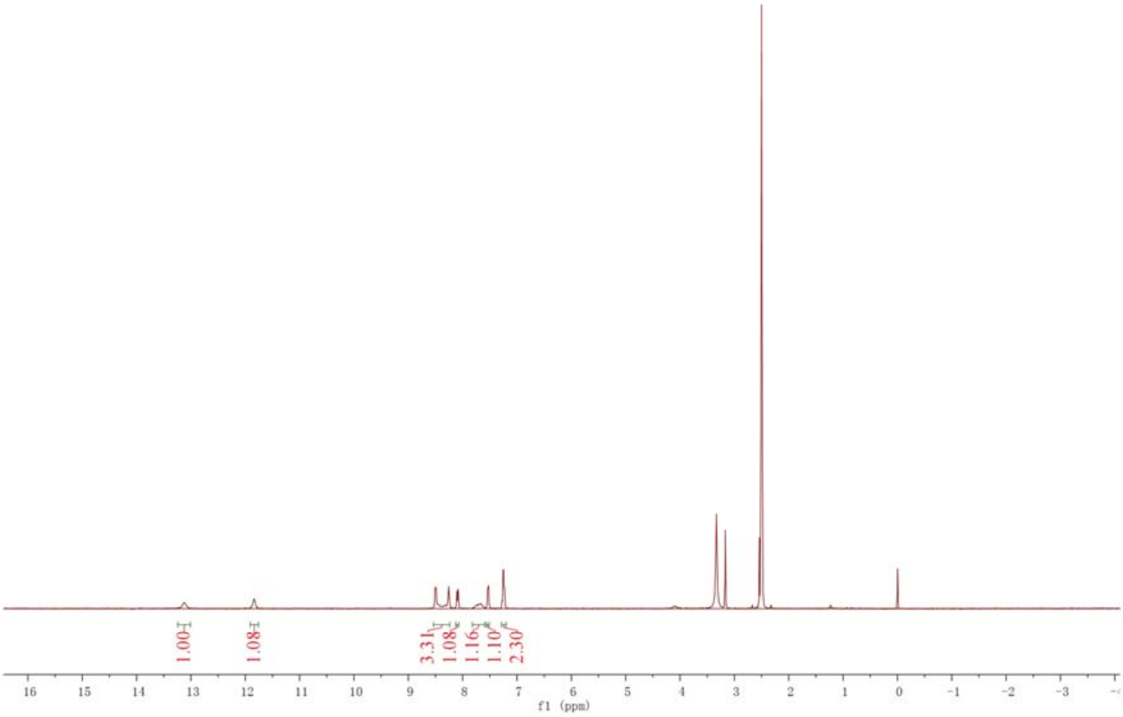

Figure S38. ^{1}H-NMR of AI-17.

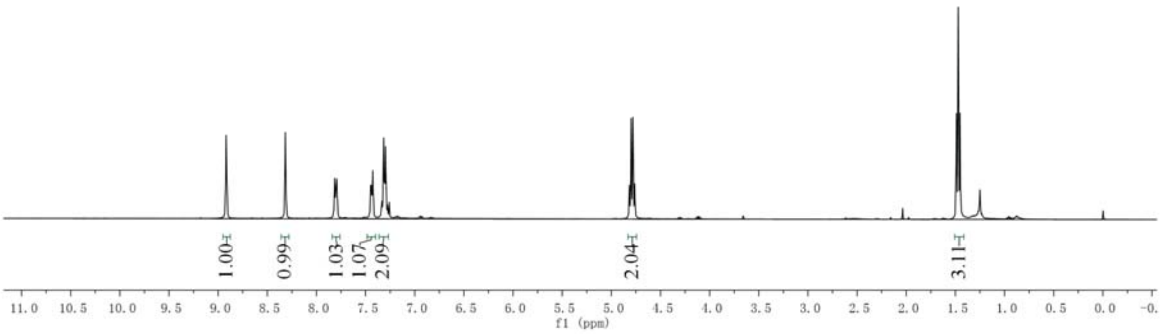

Figure S39. ¹H-NMR of AI-18.

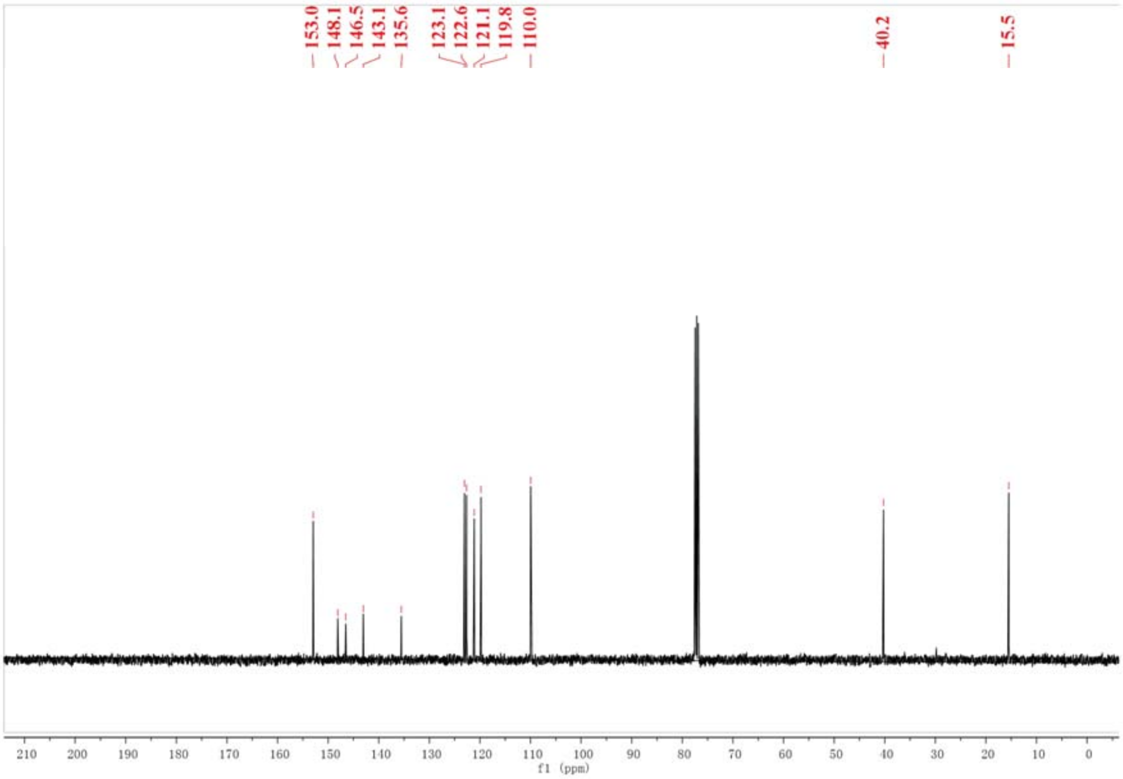

Figure S40. 13C-NMR of AI-18.

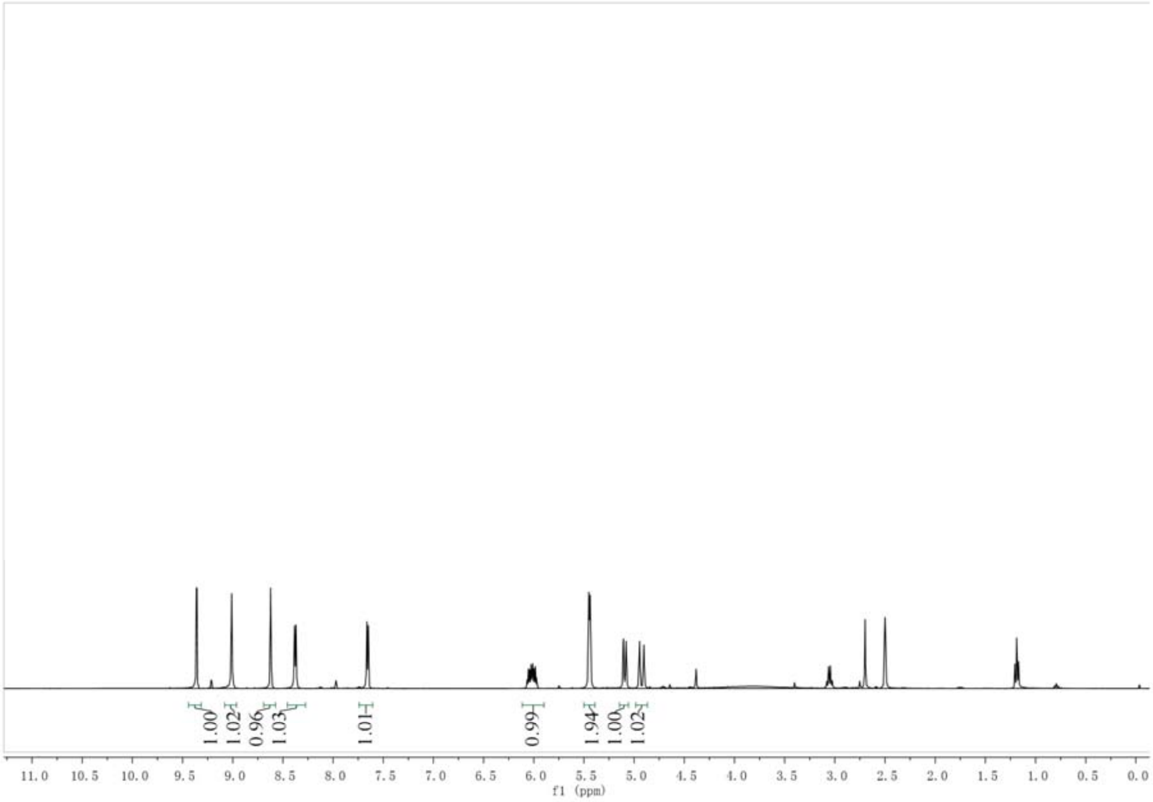

Figure S41. ^{1}H-NMR of AI-19.

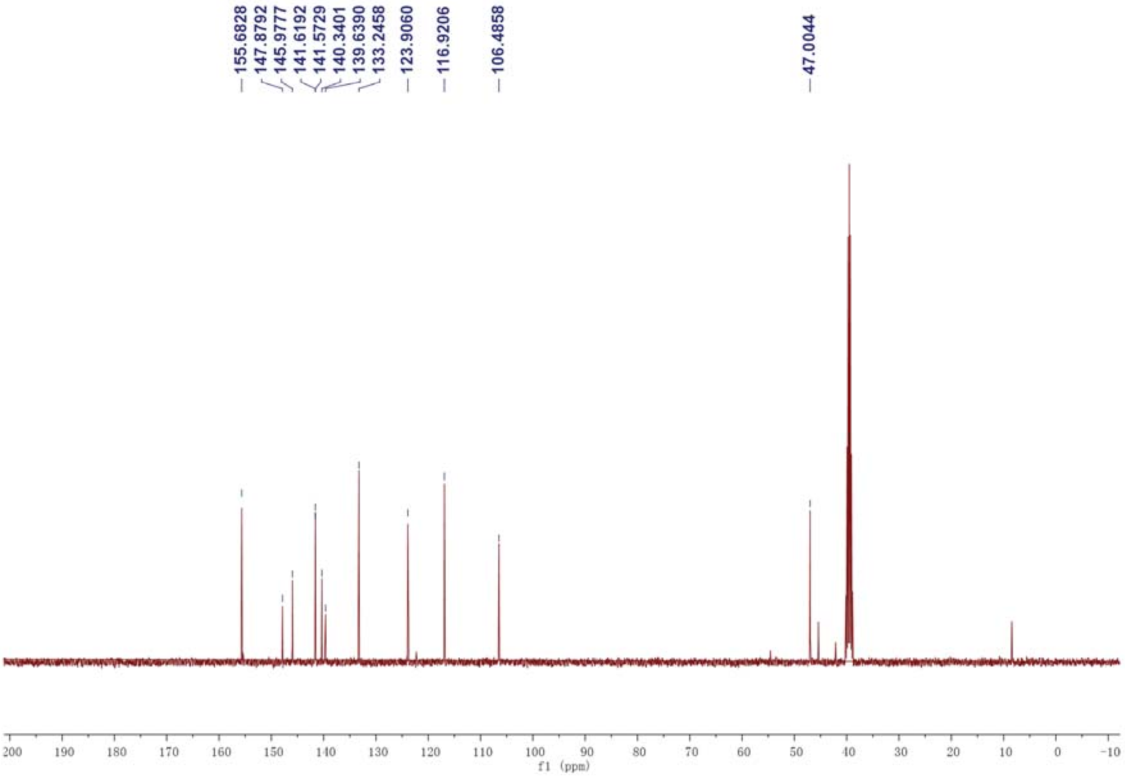

Figure S42. ^{13}C-NMR of AI-19.

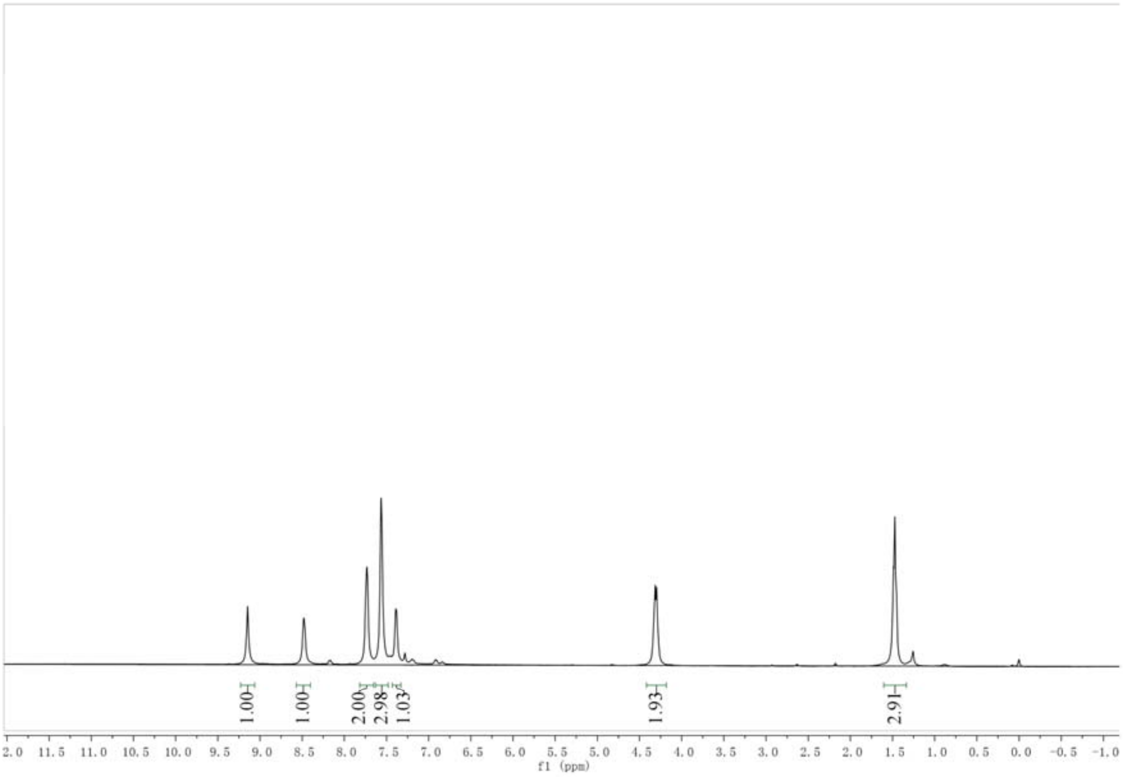

Figure S43. ^{1}H-NMR of AI-20.

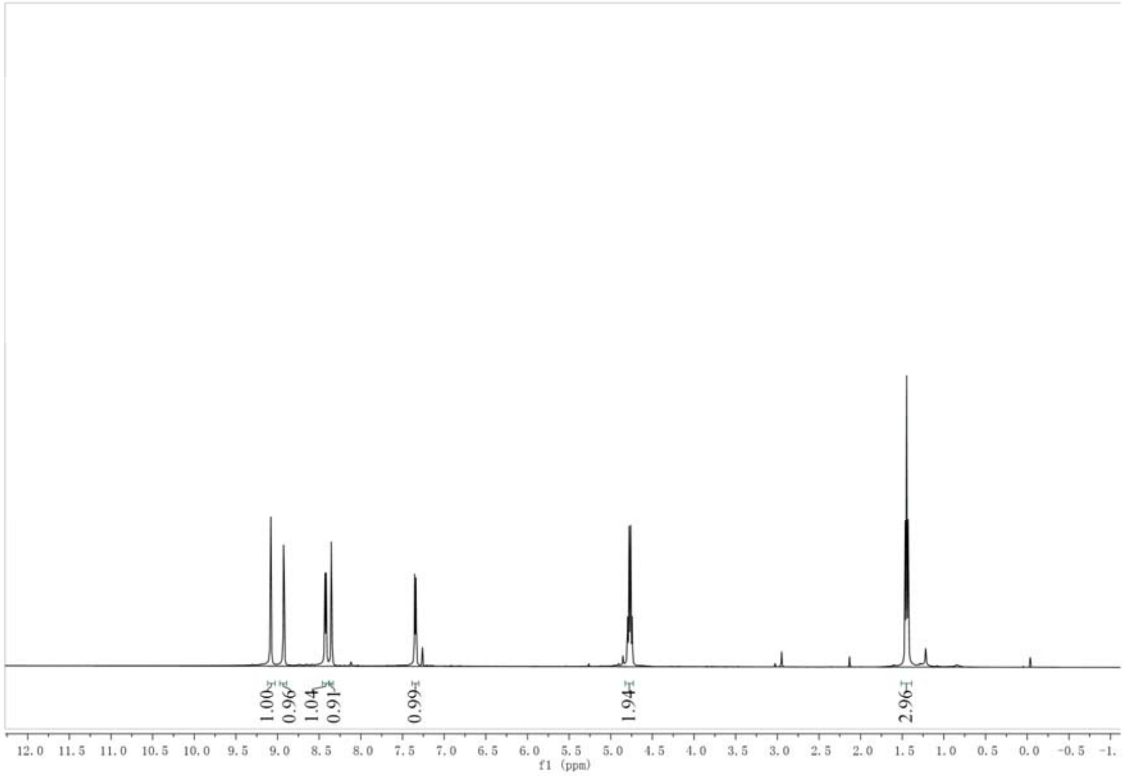

Figure S44. ^{1}H-NMR of AI-21.

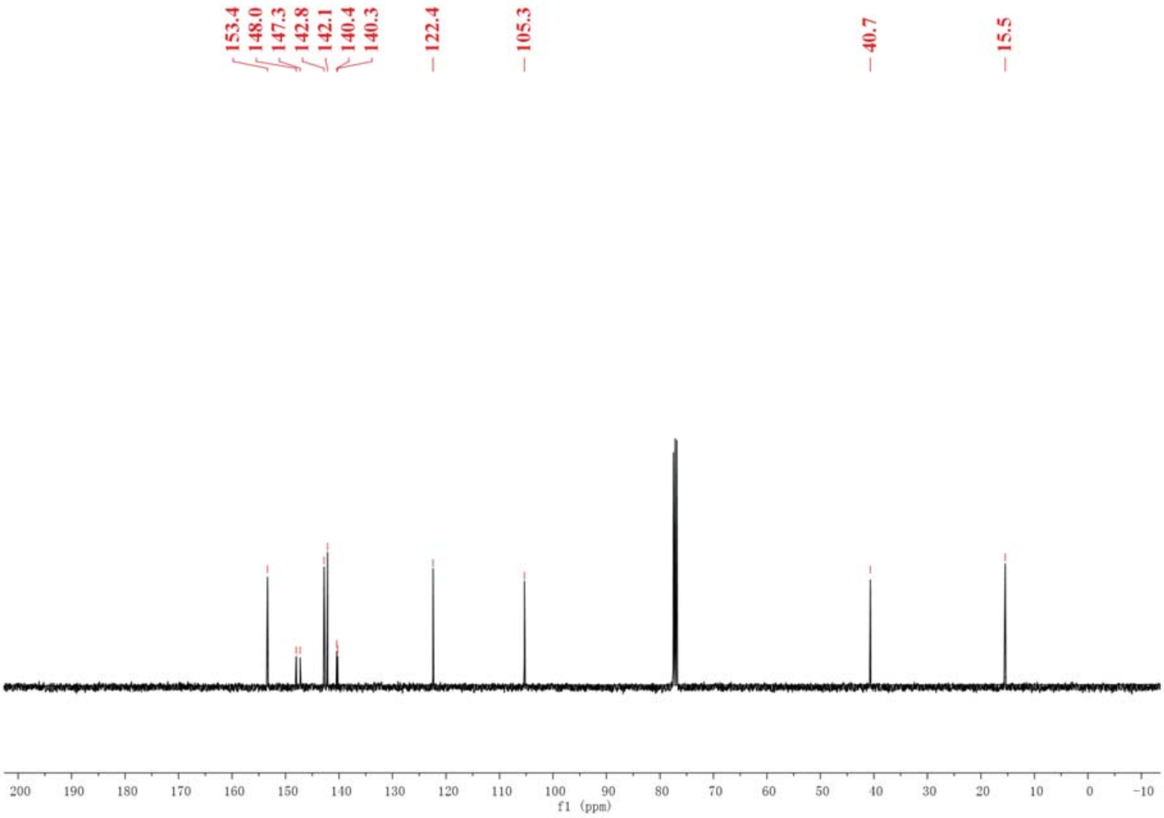

Figure S45. ^{13}C-NMR of AI-21.

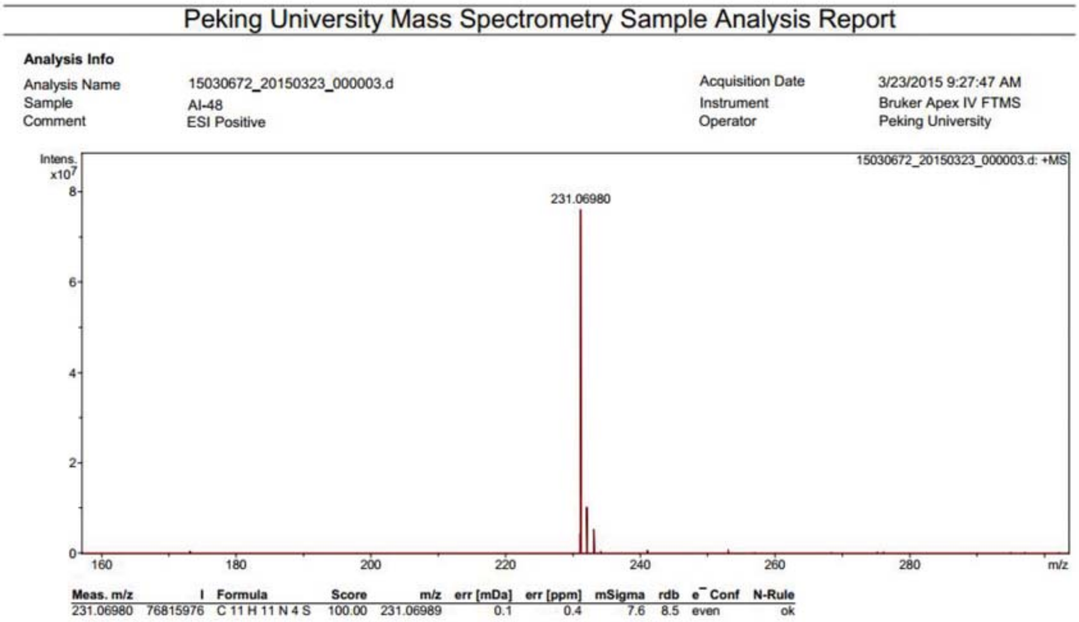

Figure S46. HR-EI-MS spectra of AI-21.

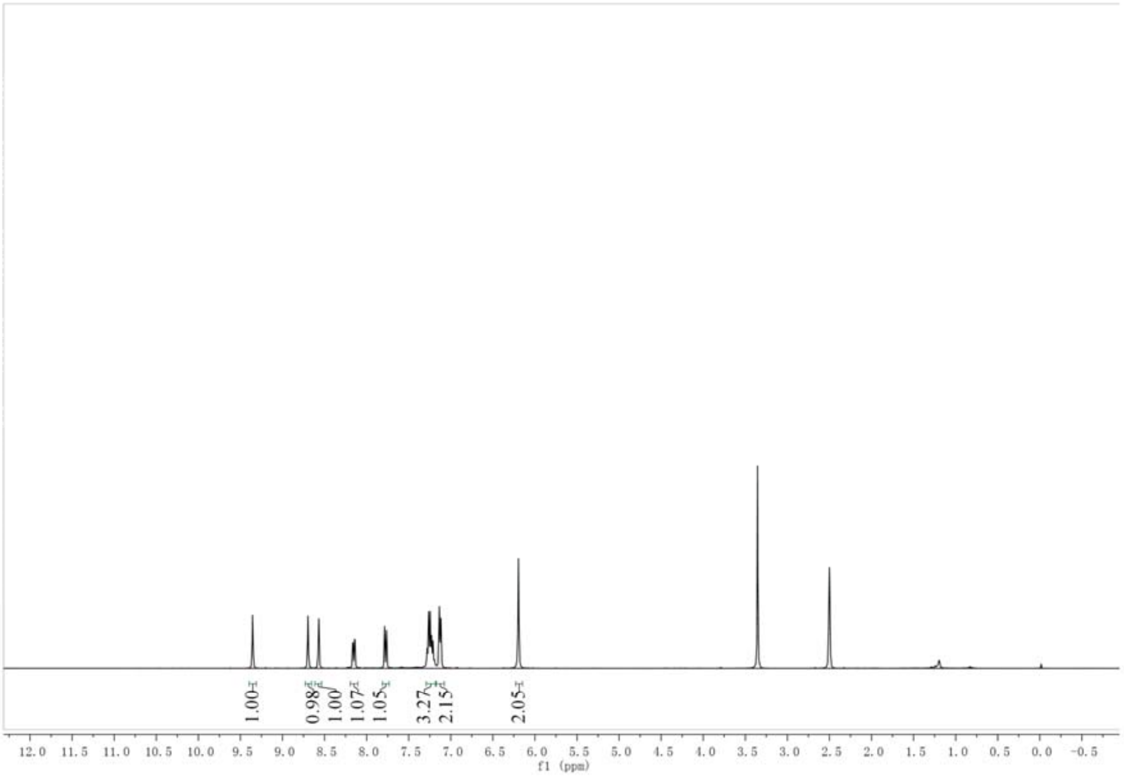

Figure S47. ^{1}H-NMR of AI-22.

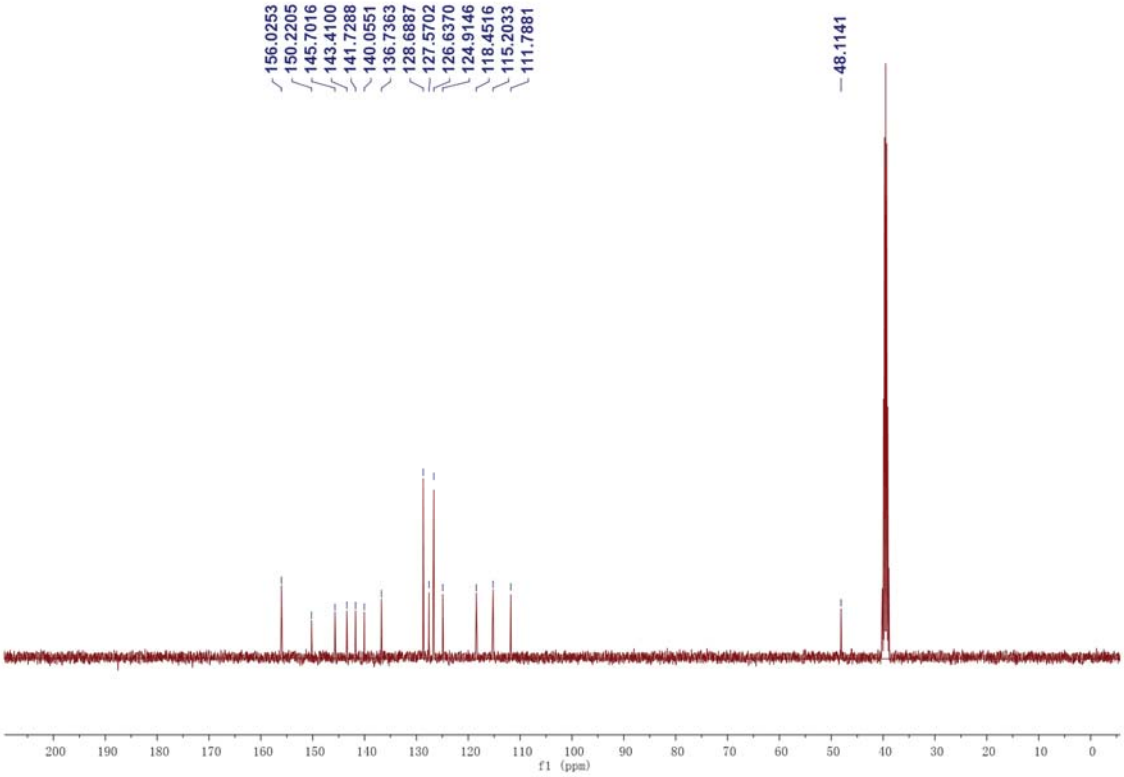

Figure S48. ^{13}C-NMR of AI-22.

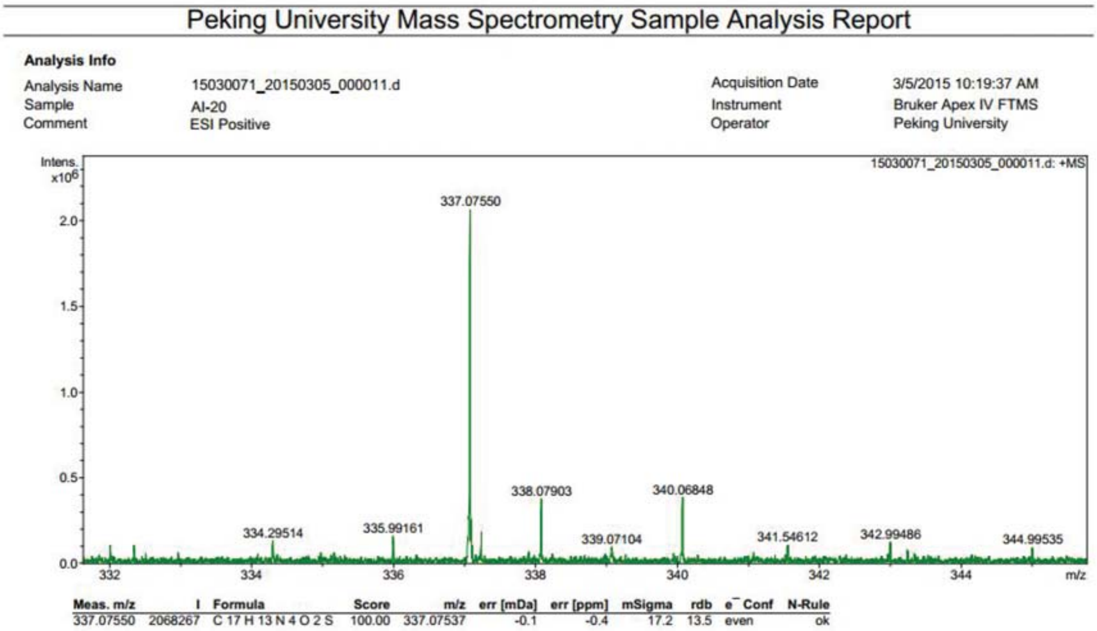

Figure S49. HR-EI-MS spectra of AI-22.

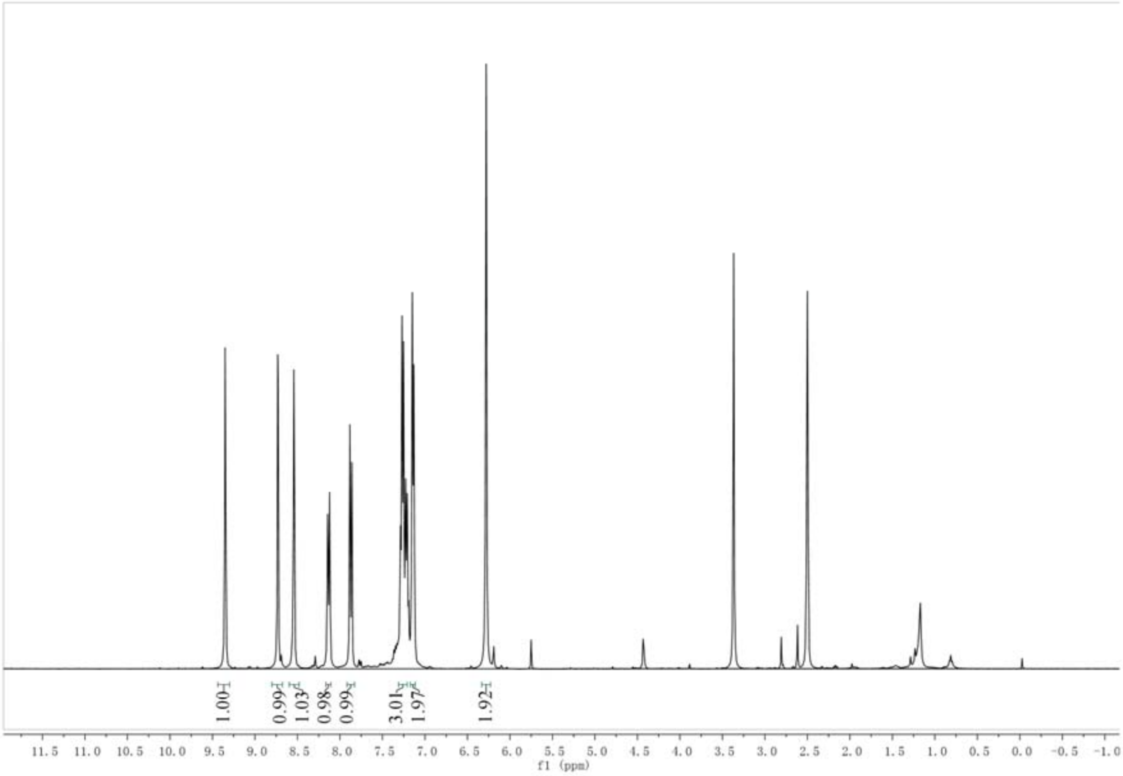

Figure S50. ^{1}H-NMR of AI-23.

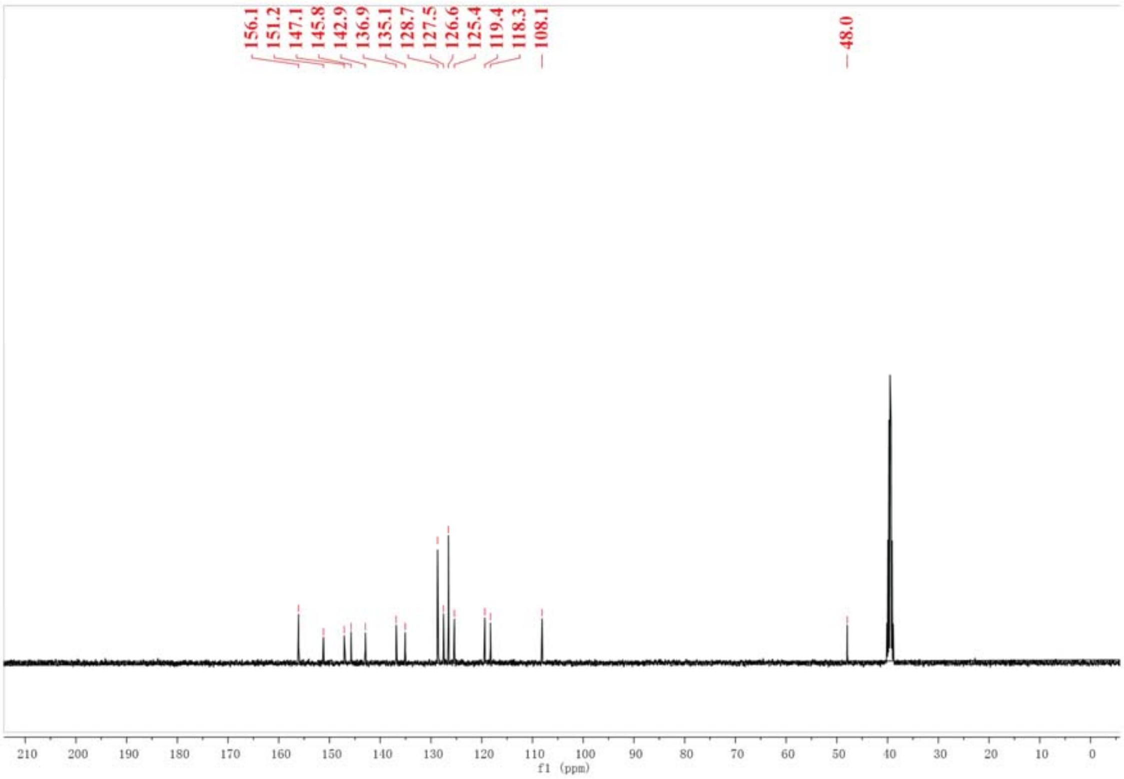

Figure S51. ^{13}C-NMR of AI-23.

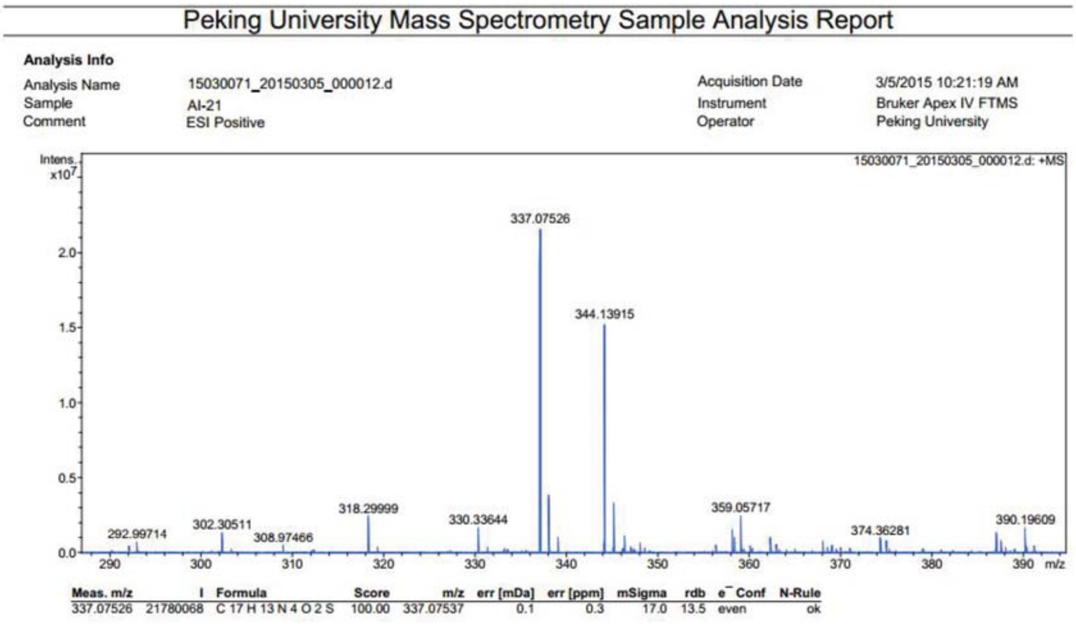

Figure S52. HR-EI-MS spectra of AI-23.

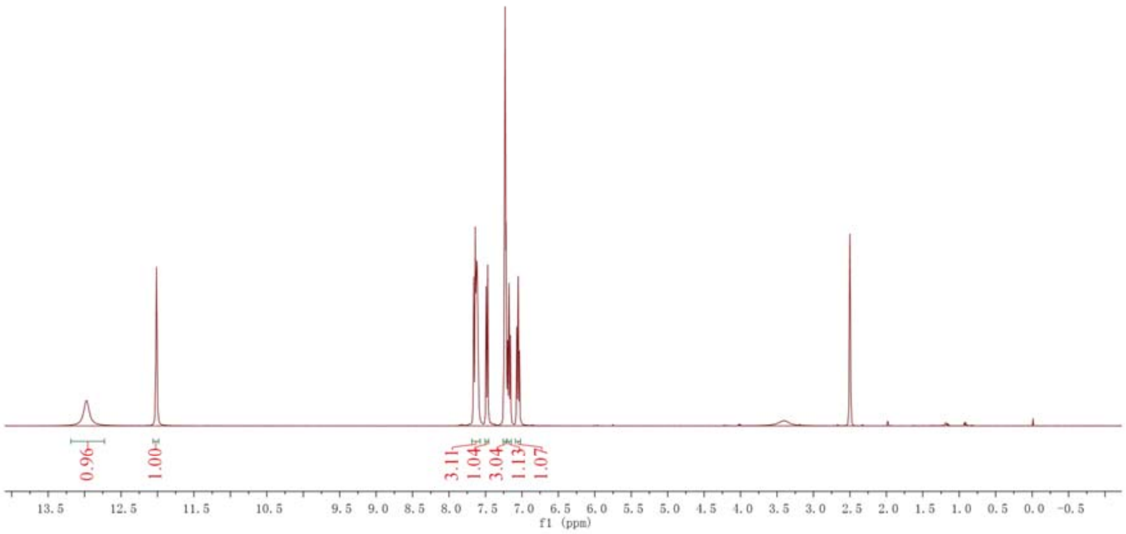

Figure S53. ^{1}H-NMR of AI-24.

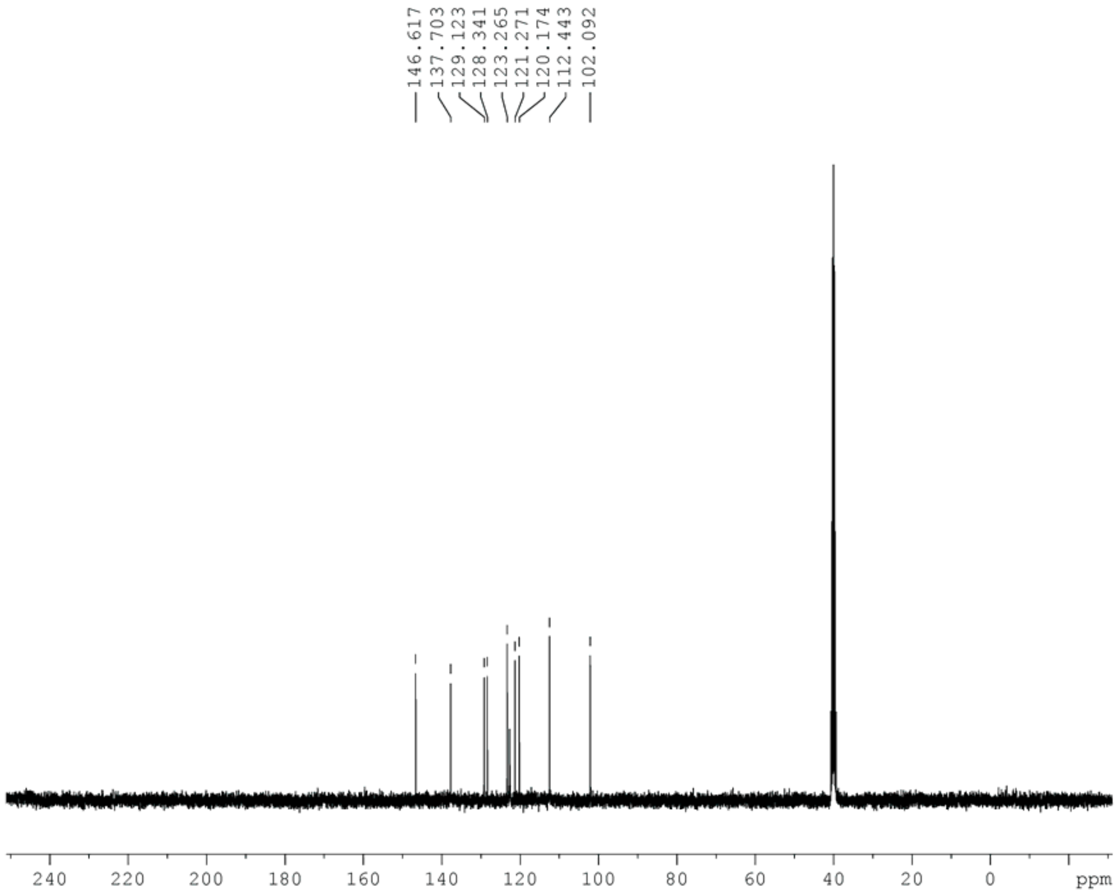

Figure S54. ^{13}C-NMR of AI-24.

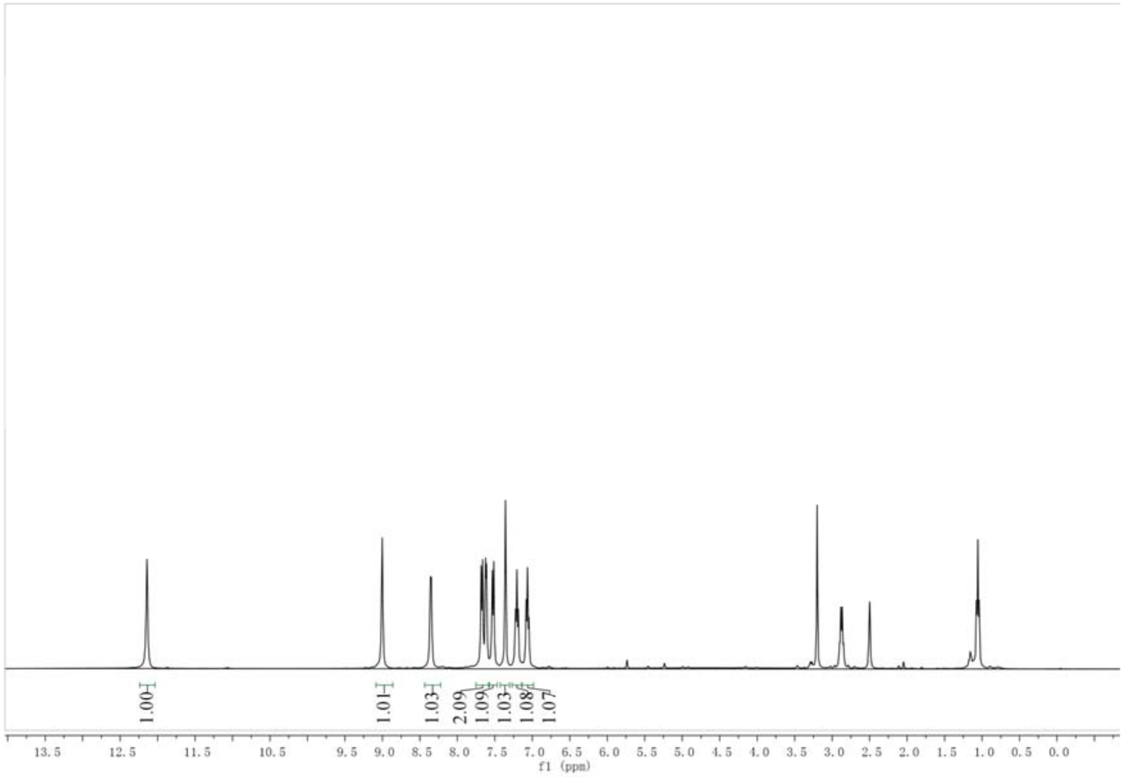

Figure S55. 1H-NMR of AI-25.

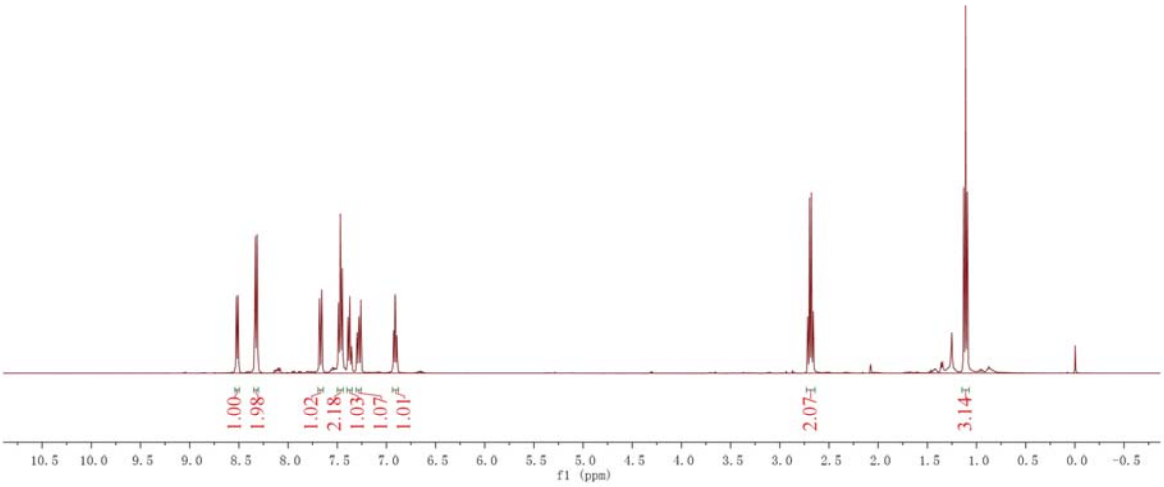

Figure S56. ^{1}H-NMR of AI-26.

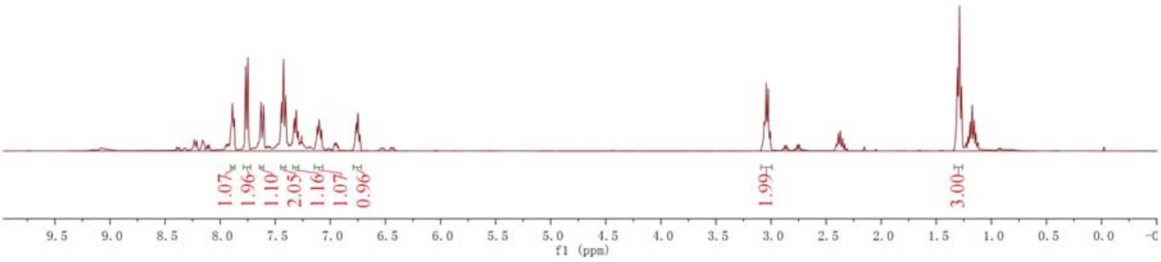

Figure S57. ^{1}H-NMR of AI-27.

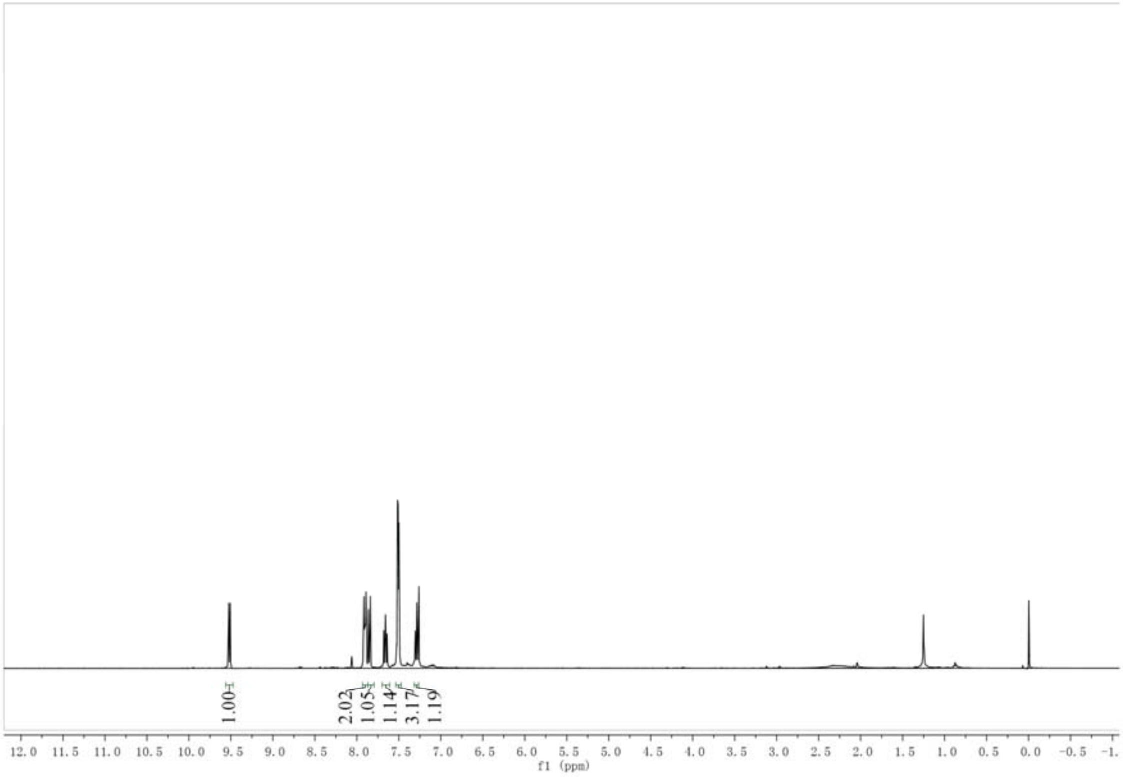

Figure S58. ^{1}H-NMR of AI-28.

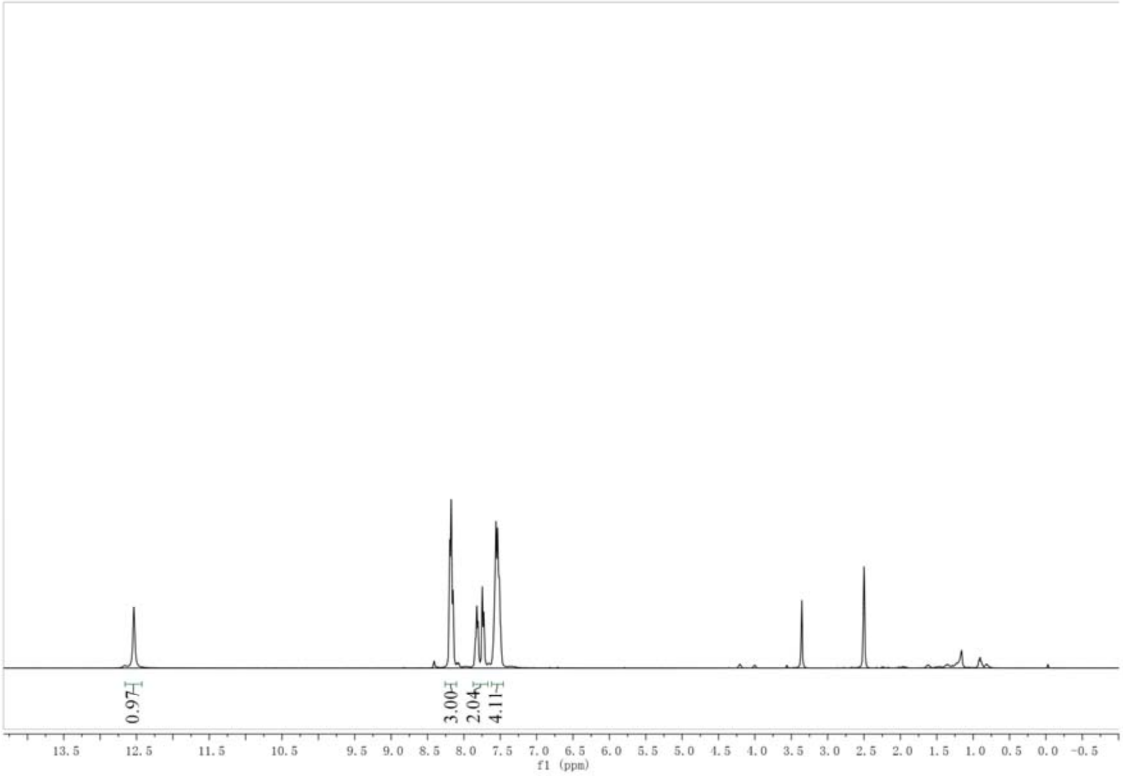

Figure S59. ^{1}H-NMR of AI-29.

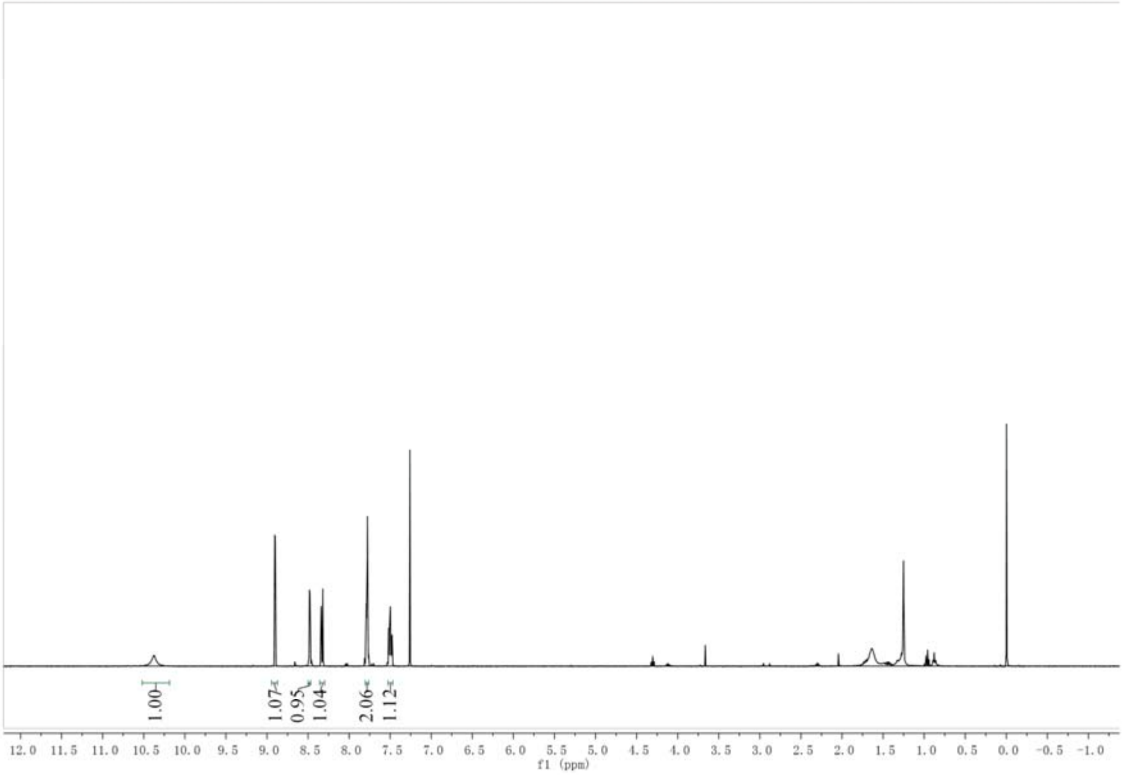

Figure S60. ^{1}H-NMR of AI-30.

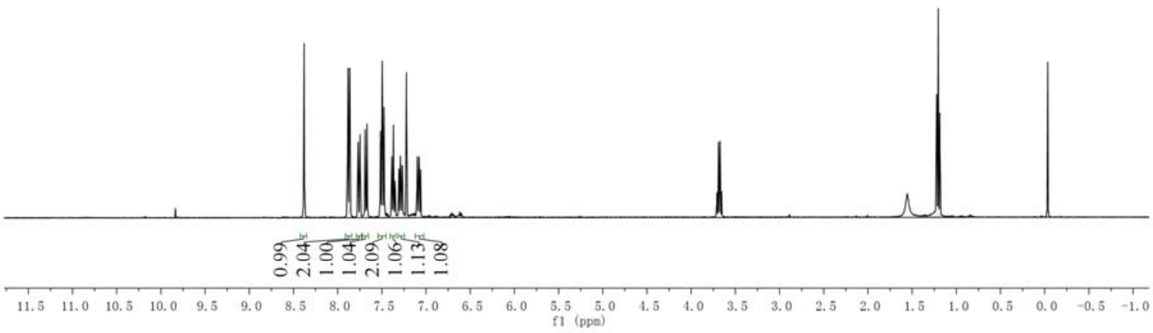

Figure S61. ^{1}H-NMR of AI-31.

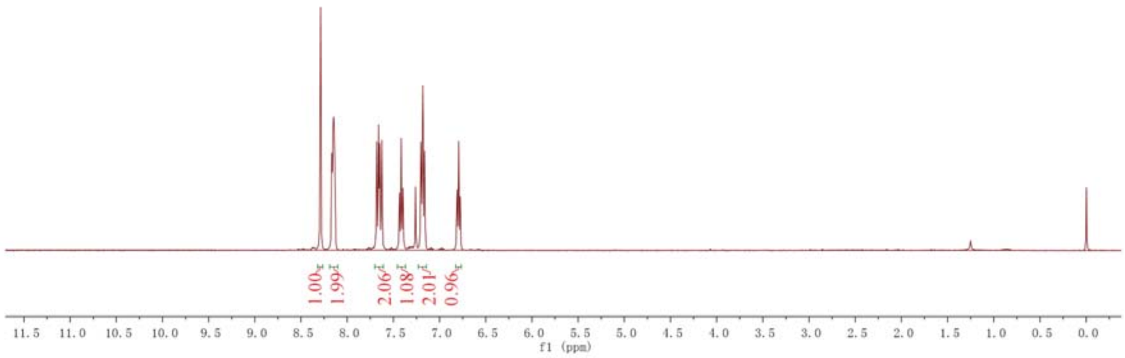

Figure S62. ^{1}H-NMR of AI-32.

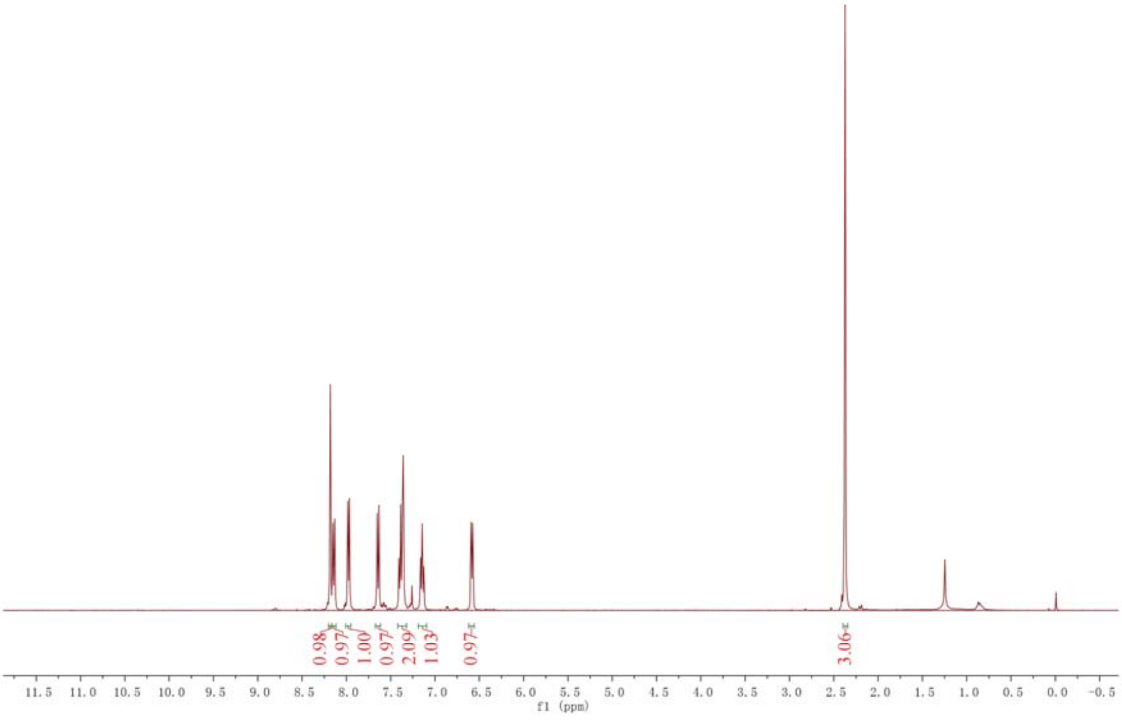

Figure S63. ^{1}H-NMR of AI-33.

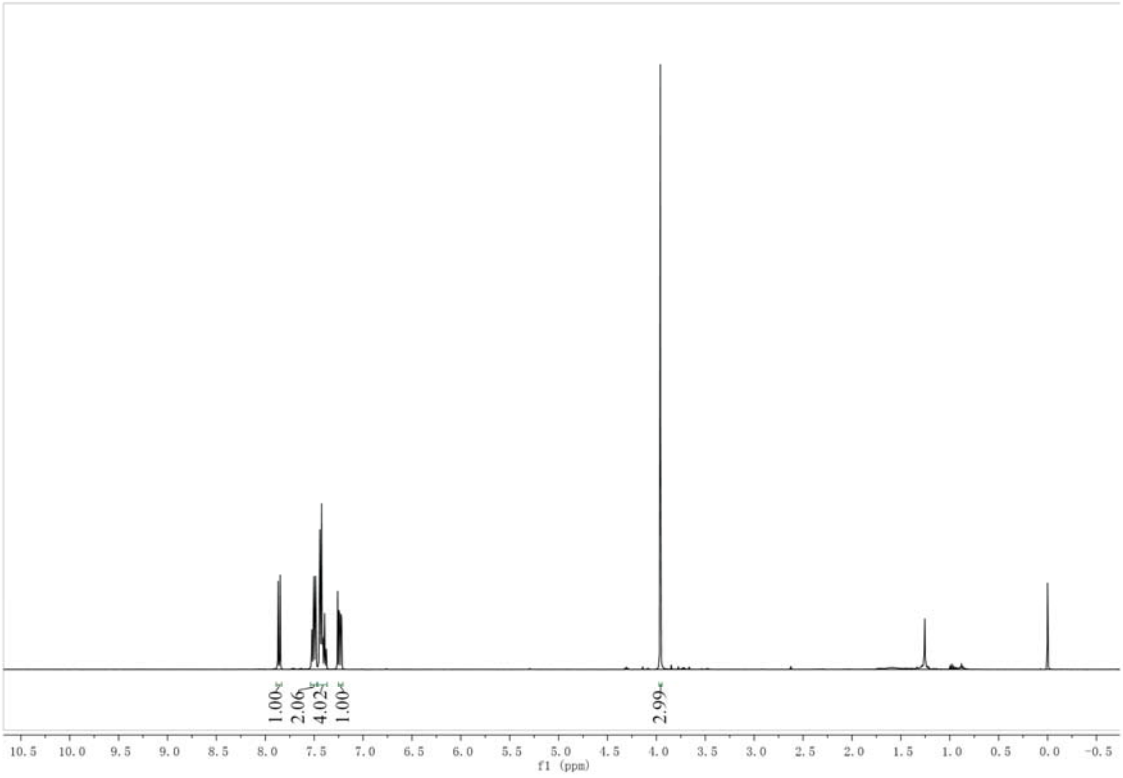

Figure S64. ^{1}H-NMR of AI-34.
